# *Irigenin*, a novel lead from Western Himalayan chemiome inhibits Fibronectin-Extra Domain A induced metastasis in Lung cancer cells

**DOI:** 10.1038/srep37151

**Published:** 2016-11-16

**Authors:** Asif Amin, Naveed Anjum Chikan, Taseem A. Mokhdomi, Shoiab Bukhari, Aabid M. Koul, Basit Amin Shah, Fatemeh Rahimi Gharemirshamlu, Asrar H. Wafai, Ayub Qadri, Raies A. Qadri

**Affiliations:** 1 Department of Biotechnology, University of Kashmir, Srinagar (J and K), 190006, India; 2 Hybridoma Laboratory, National Institute of Immunology, New Delhi, 110067, India; 3 Aaidah Life Sciences Pvt. Ltd., New Delhi, 110025, India; 4 Molecular Reproduction, Development & Genetics Lab, Indian Institute of Science, Bangalore, 560 012, India; 5 Department of Biology, Islamic Azad University, Kāzerūn, Iran

## Abstract

Several lines of evidence indicate that Fibronectin Extra Domain A (EDA) promotes metastatic capacity of tumor cells by engaging cell surface α9β1 integrins. This interaction mediated by the C-C loop of EDA activates pro-oncogenic signaling pathways leading to epithelial to mesenchymal transition (EMT) of tumor cells, thus signifying its importance in control of metastatic progression. In this context the present study was designed to explore the active compounds from selected ethno-medicinal plants of western Himalayan region for targeting EDA of Fibronectin in lung carcinoma cells. Structure based informatics for drug designing and screening was employed to generate a lead compound(s) feed that were conformationally and energetically viable. Out of 120 compounds selected, *Irigenin* showed best binding-affinity with C-C loop of EDA. *Irigenin* specifically targeted α9β1 and α4β1 integrin binding sites on EDA comprising LEU46, PHE47, PRO48, GLU58, LEU59 and GLN60 in its C-C loop as evaluated by energy decomposition per residue of *Irigenin*–EDA complex. *In-vitro* cell motility assays complemented with EDA knock-in and knockdown assays distinctively demonstrated that *Irigenin* prevents metastatic capacity of lung cancer cells by selectively blocking EDA. The results presented thus project *Irigenin* as a lead compound to overcome Fibronectin EDA induced metastatic progression in lung carcinoma cells.

The complex interactions between tumor cells and the surrounding extracellular matrix (ECM) are now increasingly recognized as important determinants of tumor cell behavior such as metastasis. Cellular Fibronectin, an abundant ECM glycoprotein involved in various physiological processes has been shown to promote the metastatic features of tumor cells^1^,^2^. Fibronectin exists in various isoforms, generated as a result of alternative splicing of pre-messenger RNA at three distinct sites including extra domain A (EDA/EIIIA), extra domain B (EDB/EIIIB), and connecting segment III^3^. Fibronectin may occur in a soluble dimeric form found in plasma and secreted by hepatocytes or an insoluble multimeric form present within the ECM produced by fibroblasts and epithelial cells^4^. The plasma Fibronectin lacks both EDA and EDB domains while as cellular Fibronectin contains the variable proportions of EDA or EDB segments^4^,^5^.

The Fibronectin imparted metastatic behavior to tumor cells has been attributed to its EDA domain. EDA containing Fibronectin has been shown to promote metastasis and vasculogenesis in a wide variety of cancer types^6^,^7^,^8^. In lung and Colon cancers, the role of EDA is becoming increasingly clear and Fibronectin containing EDA has been found to induce cell spreading and migration, thus pointing to its role in metastasis^9^. The EDGIHEL peptide comprising the C-C loop within the EDA facilitates its binding to α9β1 and α4β1 integrins^10^. The indispensability of the C-C loop for integrin binding has been demonstrated by blocking experiments using EDA-specific mAbs such as IST-9 and 3E2 which compromised the binding of EDA to α9β1 and α4β1 integrins and thus demonstrated that the C-C loop acts as a ligand for integrins^10^,^11^. Intriguingly, EDA within the cellular Fibronectin has been shown to promote metastasis through α9β1integrin mediated activation of pro-oncogenic signaling pathways with consequent repression of epithelial cells markers and the induction of a mesenchymal phenotype, a process referred to as Epithelial-Mesenchymal Transition (EMT)^12^. Thus the C-C loop region of EDA can be considered as a defining factor facilitating the progression of human cancers and hence may serve as an attractive target for therapeutic intervention.

In consonance with previous studies, relying upon the use of antibody or synthetic peptide based cancer treatments for targeting EDA^11^,^13^,^14^, we used an alternate approach based on bioactive compounds derived from natural sources to target EDA. Natural products acting as chemopreventive agents serve as alternative and safer cancer treatments and constitute the major sources of currently available anti-cancer drugs^15^. In this study, the bioactive compounds from commonly used medicinal plants of western Himalayan region were screened for their abilities to overcome the metastasis of lung carcinoma cells. The plant products from western himalayan region have been traditionally used for their diverse pharmacological properties including anticancer, antimalarial, antibacterial and anti- HIV activities^16^.

The selected bioactive compounds were screened for their affinities against C-C loop region of EDA employing computer aided drug. Shortlisted compounds were further channeled for Lipinski Rule of five^17^, predicted mutagenicity or carcinogenicity, and Absorption Distribution Metabolism Excretion (ADME) properties^18^. Four compounds retrieved from the above procedure were later assayed for their cytotoxic/anti-proliferative activity under *in vitro* conditions. Among four natural compounds, *Irigenin* demonstrated the better efficacy in terms of inhibiting the rate of cancer cell proliferation as compared to other shortlisted compounds. This led us to study the compound complex of *Irigenin* and EDA at atomic level using Molecular Dynamics simulation for being able to observe the complex under virtual microscope. Finally, we demonstrated that *Irigenin* inhibits the migration and invasion of lung carcinoma cells by modulating EMT.

## Results and Discussion

### Screening of natural compounds based on favorable molecular interactions with EDA

**Figure 1:**
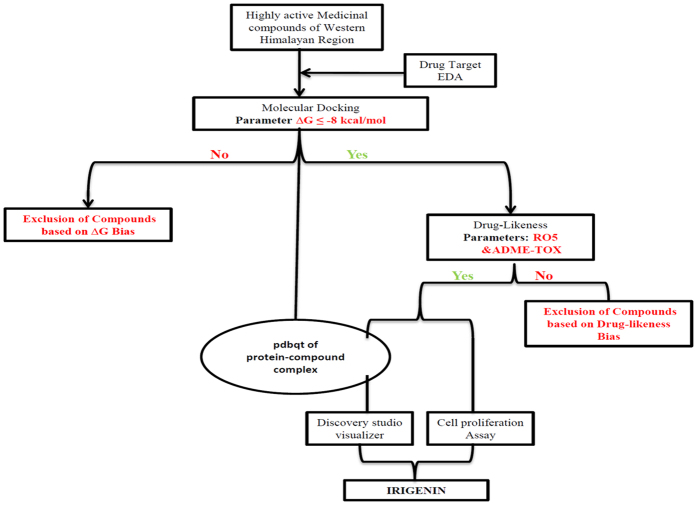
The *modus operandi* used to limit the number of compounds involves two main limiting steps: First limitation was done on the basis of binding energy and the second using drug-likeness parameters.

The strategy laid down to screen the natural compound library is depicted in Fig. 1, wherein, firstly Molecular docking simulations were employed to the NMR structure of EDA (PDB ID: 1J8K) with 120 bioactive compounds of western Himalayan region (Table S1, ESI†) and those compounds with Gibbs free energy of ≤−8 kcal/mole were selected (42 compounds) and subjected further to 3 step limiting bias. The first bias was set for carcinogenicity and mutagenicity prediction that yielded only 5 compounds out of 42 entitled for the next screening step, which fundamentally is based on ‘rule of five’ (RO5) parameters. The permissible range of RO5 includes molecular weight ≤500 Dalton, clog p (lipophilicity) ≤4.5, Hydrogen bond donors ≤5 and Hydrogen bond acceptors ≤10. Compounds which follow the RO5 are known to have better absorption or permeability across the biological membranes and 90% of orally active drugs that have achieved phase II clinical status are in agreement with this mnemonic^17^. We observed that only 4 compounds viz. *Emodin, Irigenin, Tectorigenin* and *Safranal* out of 5 qualified RO5 parameters and therefore these compounds were channeled further for prediction of ADME properties (Table S2, ESI†). The four complexes of aforementioned compounds with EDA were studied using discovery studio visualizer (Table S3, ESI†). All the compounds were found to jam C-C loop site by non covalent interaction (hydrogen bond). *Irigenin* (chem. ID 5464170) was found to have a ΔG of −10.04 Kcal/mol. The binding pocket of this molecule in the C-C loop comprised of GLY42, ILE43, PHE47, GLU45 and HIS44. It forms six hydrogen bonds with the binding domain of EDA including four hydrogen bonds with HIS44 and ILE43 (1.81–2.43 Å). Oxygen at 3^rd^, 4^th^, 6^th^ and hydrogen at 33^rd^ position of *Irigenin* forms these hydrogen bonds. *Safranal* (chem. ID 61041), forms single hydrogen bond with TYR36 of EDA with a distance of 1.99 Å. The binding pocket of this compound comprises of HIS44, TYR68, LEU46, GLY61, TYR36, LEU62 and LEU59. It forms a complex with a ΔG of −9.29Kcal/mol. *Emodin* (chem. ID 3220) binding pocket comprises of ASP41, GLY42, ILE43, GLU45 and HIS44. The interaction has a ΔG of −8.43 Kcal/mol and includes three hydrogen bonds, among which a hydrogen bond between its 2-O position and Ile43 of EDA with a distance of 2.37 Å. *Tectorigenin* (Chem. ID 5281811) forms three hydrogen bonds with EDA, with a ΔG of −8.23 Kcal/mol. which is least of the four shortlisted compounds. ILE43 of the EDA forms two hydrogen bonds with *Tectorigenin*, the oxygen at 3^rd^ and hydrogen at 29^th^ position are the atoms of the *Tectorigenin* that are involved in h-bond formation with a bond length of 2.05 Å and 2.12 Å respectively. Thus the data generated depicted *Irigenin* (chem. ID 5464170), a flavanoid from *Iris kashmiriana*, as the lead compound with ΔG score of −10.04 Kcal/mol.

### Effect of shortlisted compounds on proliferation of model human lung carcinoma cells

After the initial *in-silico* screening, all the 4 shortlisted compounds were further evaluated for anti-proliferative activities against model human lung cancer cell lines, A549 and NCI-H522 by MTT assay. These cell lines were found to constitutively express Fibronectin isoform containing EDA and therefore were selected to test the effect of these compounds (Figure S1). Out of the 4 compounds tested, *Irigenin* emerged as the best compound, showing relatively pronounced effect on growth inhibition of A549 and NCI-H522 cells with IC_50_ value of 58 μM and 61 μM in A549 and NCI-H522 respectively (Figure S2). The results demonstrated that the proliferative abilities of these cells decreased in the presence of *Irigenin* in a dose-dependent manner (Figure S2). Collectively, the data projected *Irigenin* as a candidate compound for further evaluation.

### *Irigenin* inhibits migration and invasion of human Lung carcinoma cells

**Figure 2:**
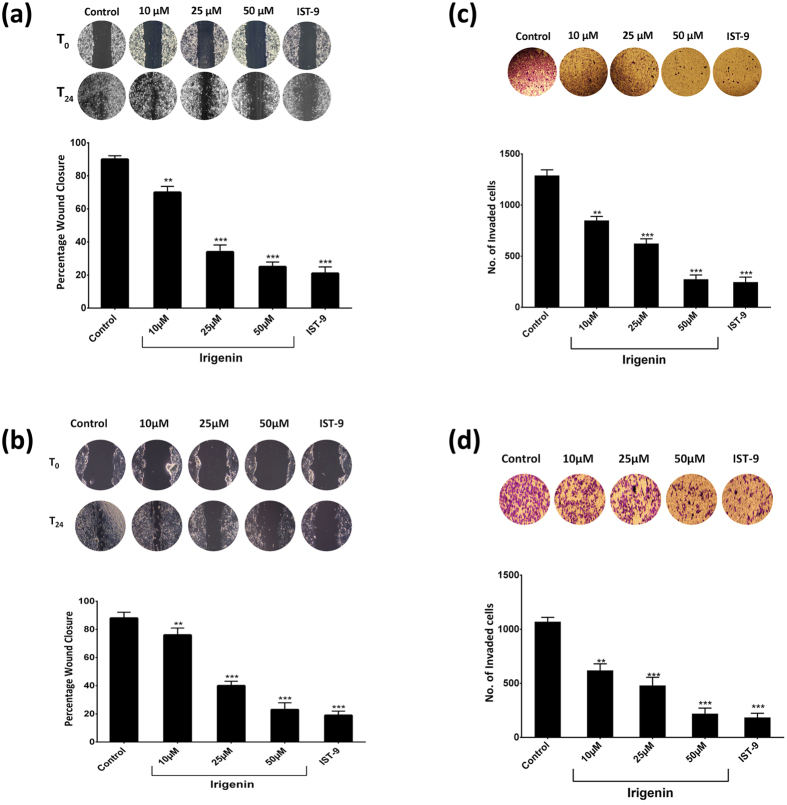
Effect of *Irigenin* on the migration and invasiveness of A549 and NCI-H522 cells. *Wound healing assay:* (**a**) A549 Cells or (**b**) NCI-H522 cells were grown to confluency, wounded and then grown in presence of 20 μg/ml of EDA neutralizing antibody (IST-9) or indicated concentrations of *Irigenin* or DMSO (control) for 24 h. The Scratched areas were photographed at zero hour (T = 0) and then subsequently later at 24 h (T = 24). *Transwell Invasion assay:* (**c**) A549 Cells or (**d**) NCI-H522 cells were placed in matrigel coated transwell chambers in the presence of 20 μg/ml of EDA neutralizing antibody (IST-9) or indicated concentrations of *Irigenin* or DMSO (control) and allowed to invade. After 24 h, the invaded cells were fixed, stained, photographed and counted. Data represented as mean ± SD of results obtained from at least three independent experiments for both the assays. **p < 0.01; ***p < 0.001 compared to control.

Cellular motility and invasiveness are indispensible to a complex and multistep process of cancer metastasis. Cellular Fibronectin containing EDA has been found to be a potent inducer of cancer cell migration, invasion and scattering *in vitro*^9^. In order to evaluate the effect of *Irigenin* on the EDA induced metastatic behavior, EDA characterized A549 and NCI-H522 cells were grown to confluency, wounded and then treated with sub-toxic concentrations (10, 25, and 50 μM) of *Irigenin* for 24 h. It was observed that *Irigenin* significantly overcame the migratory abilities of these cells in comparison to control (DMSO) as was reflected by attenuated wound healing (Fig. 2a,b). Results were presented as percentage wound closure i.e., percent of the distance that the wound had closed at 24 h relative to 0 h time point. At 24 h, about 90% of the initial gap had closed in DMSO (control) treated cells, however *Irigenin* treatment showed the inhibition of wound closure in a concentration dependent manner. While about 70% of the gap had closed using 10 μM concentration, treatment with 25 and 50 μM concentrations of *Irigenin* restricted wound healing to about 35% and 25% respectively in case of A549 cells. In NCI-H522 cells, about 75% of healing was observed using 10 μM concentration of *Irigenin* which decreased to 40% and 23% wound closure using 25 μM and 50 μM concentrations respectively. Subsequently, the effect of *Irigenin* on the invasion of A549 and NCI-H522 cells was determined by transwell invasion assay, wherein cells were treated with *Irigenin* and allowed to invade the matrigel coated matrices. The results showed that *Irigenin* significantly inhibited the invasiveness of A549 and NCI-H522 cells as compared to control (Fig. 2c,d). The invasive capacity of *Irigenin* treated-cells was inhibited by 80% and 35% in case of A549 cells and 80% and 40% in NCI-H522 cells at highest and lowest non-cytotoxic concentrations of *Irigenin* respectively, compared to the control. The finding thus depicts that the two cell lines responded in a similar fashion to *Irigenin* treatment.

### *Irigenin* mediates its anti-metastatic effects by limiting Epithelial-Mesenchymal Transition

**Figure 3:**
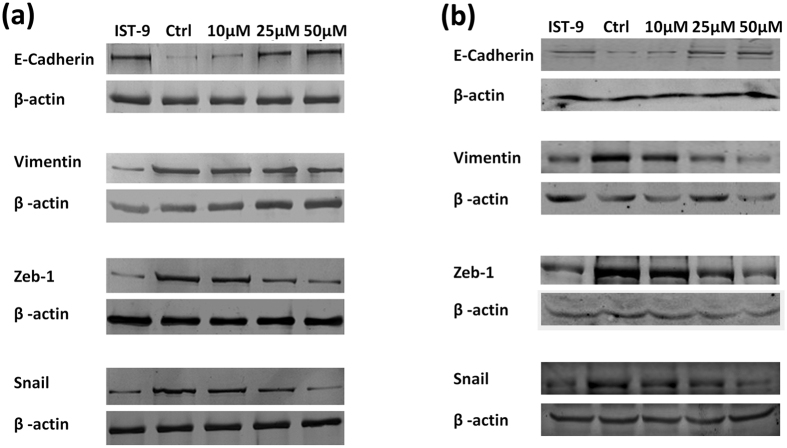
Effect of *Irigenin* on the expression of signature markers of EMT in (**a**) A549 cells (**b**) NCI-H522. Cells were cultured in the presence of 20 μg/ml of EDA neutralizing antibody (IST-9) or indicated concentrations of *Irigenin* or DMSO (control) for 24 h, followed by lysis and western blotting analysis for the expression of EMT markers. β-actin serves as loading control.

One of the key steps in tumor metastasis is the acquisition of cellular motility and invasiveness. During this process, tumor cells lose their epithelial features and acquire mesenchymal characteristics through the activation of EMT program. EDA within the cellular Fibronectin has been shown to be a defining factor promoting migration and invasion of lung carcinoma cells by inducing EMT through its interactions with α9β1 integrins^10^. Having established that *Irigenin* inhibits the migration and invasion of A549 cells, we set out to investigate whether *Irigenin* mediates its anti-metastatic effects via EMT. A549 and NCI-H522 cells were treated with sub-toxic doses of *Irigenin* and the expression of EMT markers was evaluated. Western blotting analysis showed that the treatment of cells with *Irigenin* modulates the expression of EMT markers. The expression of E-cadherin, a signature epithelial marker showed a dose dependent increase with concomitant decrease in the expression of a mesenchymal marker, vimentin. These results were further substantiated by the dose-dependent down-regulation of the EMT-associated transcription regulators, Snail and Zeb-1, upon *Irigenin* treatment in both the cell lines (Fig. 3).

### *Irigenin* doesn’t affect metastatic capacity of EDA negative T47D cells

**Figure 4:**
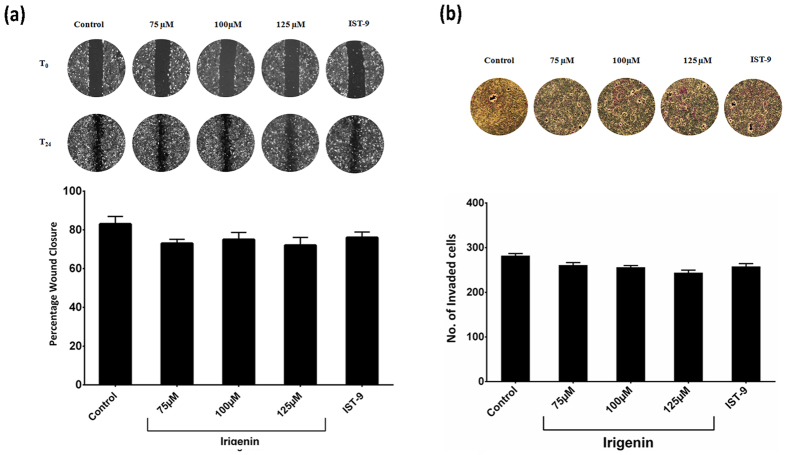
Effect of *Irigenin* on the migration and invasiveness of EDA negative, T47D cells. (**a**) Wound healing assay: T47D cells were grown to confluency, wounded and then grown in presence of 20 μg/ml of EDA neutralizing antibody (IST-9) or indicated concentrations of *Irigenin* or DMSO (control) for 24 h. The Scratched areas were photographed at zero hour (T = 0) and then subsequently later at 24 h (T = 24). (**b**) Transwell Invasion assay: T47D cells were placed in matrigel coated transwell chambers in the presence of 20 μg/ml of EDA neutralizing antibody (IST-9) or indicated concentrations of *Irigenin* or DMSO (control) and allowed to invade. After 24 h, the invaded cells were fixed, stained, photographed and counted. Data represented as mean ± SD of results obtained from at least three independent experiments for both the assays.

**Figure 5:**
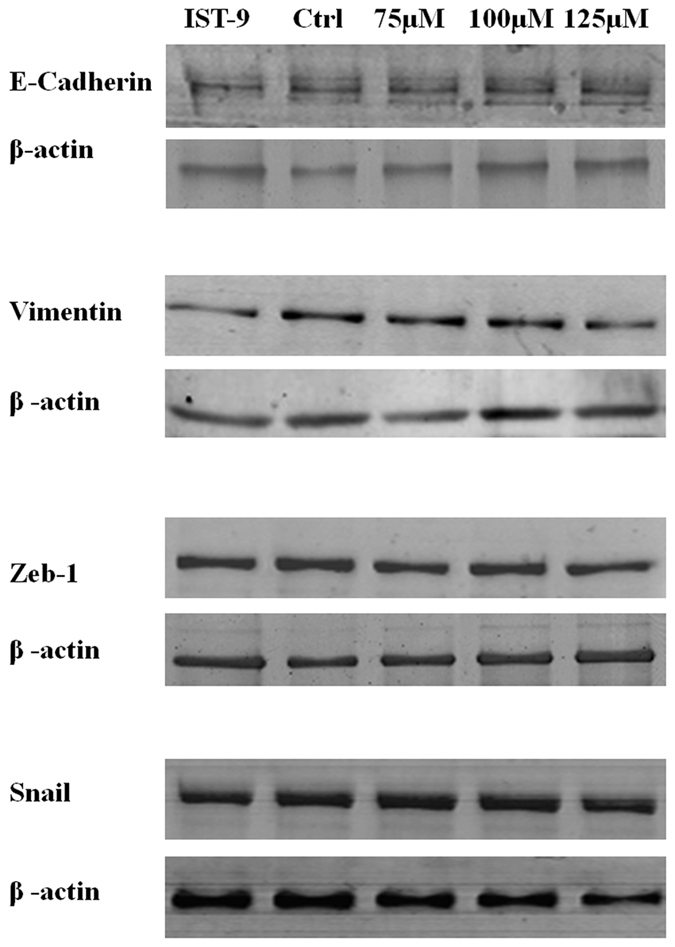
Effect of *Irigenin* on the expression of signature markers of EMT in EDA negative, T47D cells. T47D cells were cultured in the presence of 20 μg/ml of EDA neutralizing antibody (IST-9) or indicated concentrations of *Irigenin* or DMSO (control) for 24 h, followed by lysis and western blotting analysis for the expression of EMT markers. β-actin serves as loading control.

EDA within the cellular Fibronectin has been shown to promote metastasis via activation of pro-oncogenic signaling pathways with a concomitant induction of Epithelial-Mesenchymal Transition (EMT)^12^. To assess if *Irigenin* mediates its metastatic effect via EDA, T47D (breast carcinoma), a cell line which tested negative for EDA expression (Figure S1) was treated with sub-toxic doses of *Irigenin* derived alternatively by MTT assay (Figure S3). It was observed that *Irigenin* neither showed any conspicuous effect on the migration and invasion of T47D cells, nor affected expression of EMT markers even at concentrations which accounted for higher growth inhibition in A549 cells. The results were further verified by the observation that IST-9 antibody, which specifically binds EDA could not account for any observable change in T47D cell behavior (Figs 4 and 5), however, as observed treatment of A549 cells with EDA neutralizing antibody (IST-9) or *Irigenin* attenuated migratory and invasive capabilities of A549 cells (Figs 2 and 3) thus pointing towards role of EDA in ensuing *Irigenin* or IST-9 mediated EMT suppression.

### *Irigenin* selectively targets EDA to abrogate Epithelial-Mesenchymal transition

**Figure 6:**
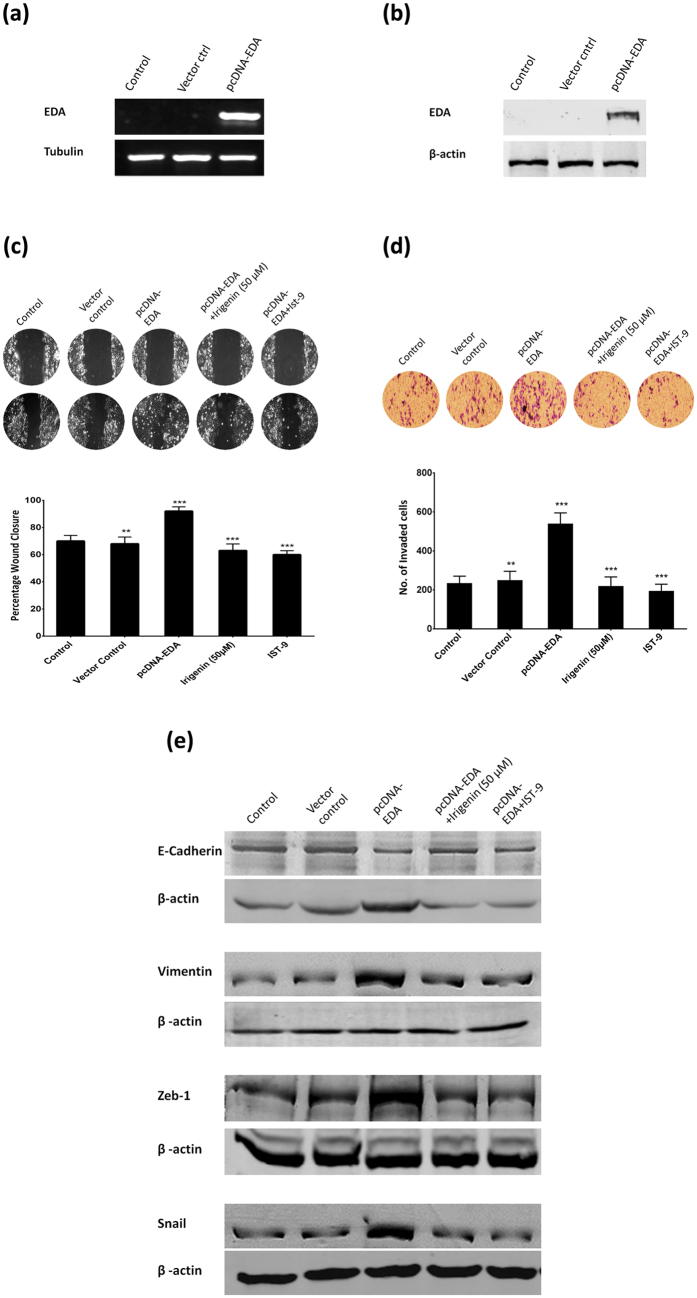
*Irigenin* overcomes EDA induced Epithelial-Mesenchymal transition. T47D cells were transfected with 2 μg pcDNA-EDA plasmid or vector control and transfection was confirmed by (**a**) Semi-quantitative reverse transcription-PCR and (**b**) western blotting. (**c**) Wound healing assay: T47D cells transfected with pcDNA-EDA were grown to confluency, wounded and then grown in presence of 50 μM *Irigenin* or 20 μg/ml IST-9 for 24 h. The Scratched areas were photographed at zero hour (T = 0) and then subsequently later at 24 h (T = 24). (**d**) Transwell Invasion assay: pcDNA-EDA transfected T47D cells were placed in matrigel coated transwell chambers in presence of 50 μM *Irigenin* or 20 μg/ml IST-9 and allowed to invade. After 24 h, the invaded cells were fixed, stained, photographed and counted. Data represented as mean ± SD of results obtained from at least three independent experiments for both the assays. **p < 0.01; ***p < 0.001 compared to control. (**e**) T47D cells transfected with pcDNA-EDA were cultured in presence of 50 μM *Irigenin* or 20 μg/ml IST-9 followed by lysis and western blotting analysis for the expression of EMT markers. β-actin serves as loading control. Cropped immunoblots are shown for comparison (full immunoblots provided in Supplementary Information).

**Figure 7:**
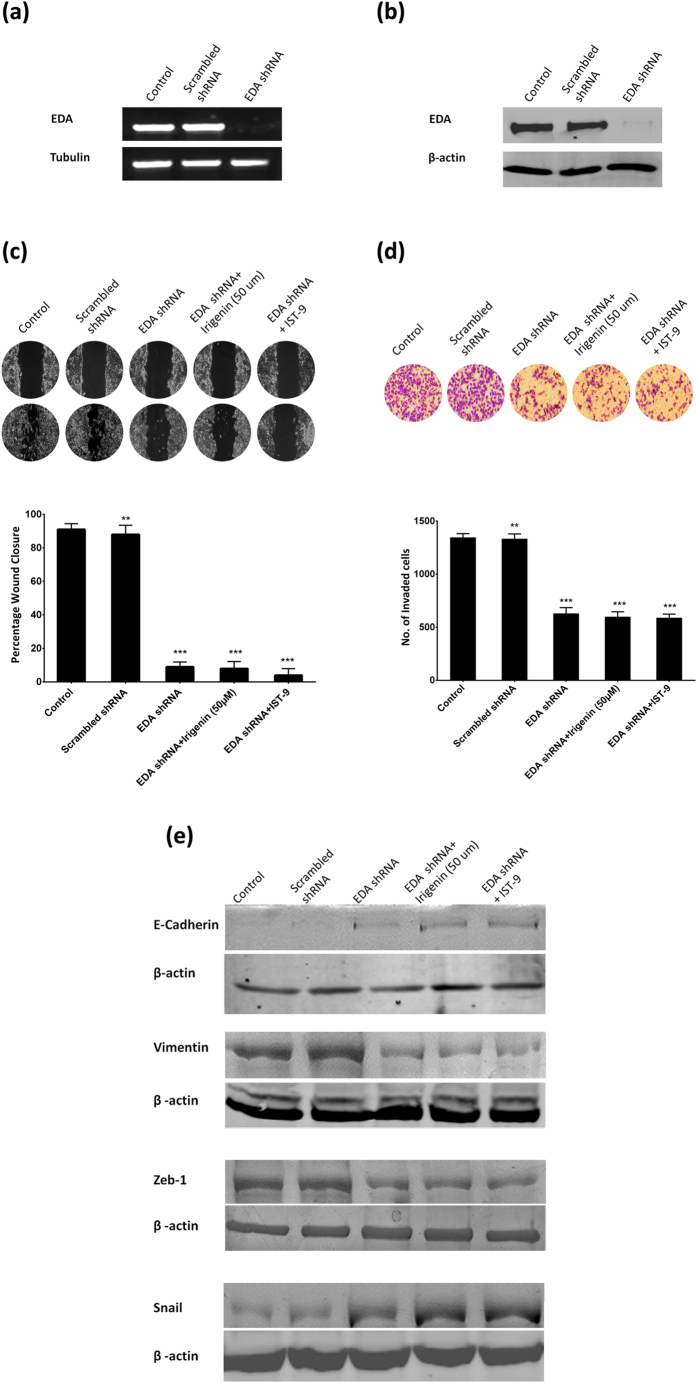
EDA Knockdown renders A549 cells unresponsive to *Irigenin* treatment. A549 cells were stably transfected with plasmid expressing Fibronectin-EDA shRNA or scrambled shRNA (mock) and silencing of EDA was confirmed by (**a**) Semi-quantative reverse transcription-PCR and (**b**) western blotting. (**c**) Wound healing assay: A549 cells transfected with Fibronectin-EDA shRNA plasmid were grown to confluency, wounded and then grown in presence of 50 μM *Irigenin* or 20 μg/ml IST-9 for 24 h. The Scratched areas were photographed at zero hour (T = 0) and then subsequently later at 24 h (T = 24). (**d**) Transwell Invasion assay: Fibronectin-EDA shRNA plasmid transfected A549 cells were placed in matrigel coated transwell chambers in presence of 50 μM *Irigenin* or 20 μg/ml IST-9 and allowed to invade. After 24 h, the invaded cells were fixed, stained, photographed and counted. Data represented as mean ± SD of results obtained from at least three independent experiments for both the assays. **p < 0.01; ***p < 0.001 compared to control. (**e**) A549 cells transfected with Fibronectin-EDA shRNA plasmid were cultured in presence of 50 μM *Irigenin* or 20 μg/ml IST-9 followed by lysis and western blotting analysis for the expression of EMT markers. β-actin serves as loading control. Cropped immunoblots are shown for comparison (full immunoblots provided in Supplementary Information).

In order to demonstrate selective inhibition of EMT by *Irigenin* via *EDA*, T47D cells (which tested EDA negative) were transfected with pcDNA-EDA construct. The confirmation of transfection was examined by checking the expression of EDA in control (non vector), vector control and EDA vector transfected T47D cells by reverse transcription-PCR and western blotting (Fig. 6a,b). Consistent with the previous reports^6^,^12^ which demonstrated induction of tumorigenesis and metastatic phenotype in EDA transfected cancer cells, it was observed that transient expression of EDA significantly increased the migration and invasiveness of T47D cells with concomitant modulation in the expression of EMT markers. These effects were however reversed by treatment with *Irigenin* or IST-9 in EDA transfected T47D cells (EDA-T4D), thus establishing selective action of *Irigenin* via targeting EDA (Fig. 6c,d,e). To further confirm the selectivity of *Irigenin* in targeting EDA, we studied metastatic behavior in EDA-knockdown-A549 cells. A549 cells were stably transfected with a plasmid encoding human Fibronectin-EDA silencing shRNA or scrambled shRNA (mock). The effect of silencing on the expression of EDA was evaluated by reverse transcription-PCR and western blotting (Fig. 7a,b). Interestingly, silencing of EDA impaired the ability of A549 cells towards migration and invasion while simultaneously favoring epithelial phenotype (Fig. 7c,d,e). The EDA silenced‒A549 cells showed no response to *Irigenin* or IST-9 treatment in migration, invasion or EMT assays. Together these findings specifically impress upon our proposed mechanism that *Irigenin* selectively interacts with EDA to abrogate metastatsis in lung carcinoma cells.

### *Irigenin* mediates its anti-metastatic effect by specifically and selectively blocking α9β1 and α4β1 integrins binding sites on C-C loop of EDA

**Figure 8:**
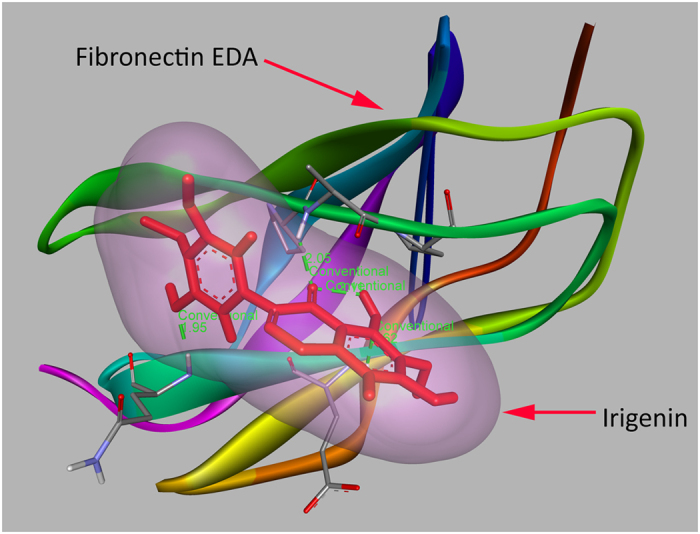
*Irigenin*-EDA complex. Binding of *Irigenin* to C-C loop of EDA depicting prominent ligand interactions.

Previous studies have demonstrated that the loop between the C and C β-strands (C-C loop) of EDA segment within Fibronectin acts as an optimal ligand binding site for α9β1 and α4β1 integrins^10^. Therefore, C-C loop of EDA was used as a putative target for drug designing. It was observed that *Irigenin* specifically targets the C-C loop of EDA (Fig. 8). The binding pocket of *Irigenin* within the C-C loop comprised of GLY42, ILE43, PHE47, GLU45 and HIS44 (Figure S4). Oxygen at 3^rd^, 4^th^, 6^th^ and hydrogen at 33^rd^ position of *Irigenin* formed 6 hydrogen bonds, including 4 hydrogen bonds with HIS44 and ILE43 of C-C loop. Intriguingly, ILE43 and HIS44 in the C-C loop constitute an indispensible part of the epitope for the mAbs that react with EDA segment of human, rat and chicken Fibronectin and block its function^11^, thus substantiating the alternative approach of targeting EDA devised in this study. Figure S4 (a), (b) and (c) shows the binding of *Irigenin* to C-C loop in different ways.

To explore the stability and binding mode of *Irigenin* with C-C loop of EDA, energy minimized *Irigenin*-EDA complex was subjected to molecular dynamics simulation for 100 ns run under GROMOS 43a1 force field. The movie of the whole run is shown in ESI† Movie, with each frame retrieved at 100 ps interval. Comparative RMSD plot showed a substantial decrease in RMSD of *Irigenin*-EDA complex throughout the 100 ns run compared to EDA alone. However, RMSD of the *Irigenin* alone remained stable at 0.1 nm (Figure S5 ESI†). Principal component analysis was employed to study the dominant motions of *Irigenin*-EDA complex. The lowest energy conformer in the Free energy landscape of *Irigenin*-EDA complex (t = 57,836 ps) was retrieved to investigate the predominant atomic interactions (Figure S6, ESI†). The hydrogen bonding pattern of this lowest energy conformer, calculated between *Irigenin*-EDA complex over time at 300 K was found to be above the average (1.8 hydrogen bonds), (Fig. 7, ESI†). Furthermore, MM-PBSA calculations were employed to evaluate the binding free energy of the complex (Table S4, ESI†). The binding energy of the simulated complex scored at −221.602 ± 35.657 kJ/mol. Figure S8 shows the free energy decomposition per residue, where the binding of *Irigenin* with the C-C loop of EDA is evident. The prominent engaging amino acids of EDA with *Irigenin* included LEU46 (−6.9 kJ/mol), PHE47 (−3.6 kJ/mol), PRO48 (−7.8 kJ/mol), GLU58 (−4.1 kJ/mol), LEU59 (−6.1 kJ/mol) and GLN60 (−3.7 kJ/mol).

## Summary and Conclusion

Fibronectin is a high molecular weight multifunctional extracellular matrix glycoprotein that plays a prominent role in tumor metastasis due to its interaction with various cellular receptors including integrins. Fibronectin exists in various isoforms, generated as a result of alternative splicing thereof due to combination of these spliced variants, corresponding to extra domain A (EDA/EIIIA), extra domain B (EDB/EIIIB), and connecting segment III (V). Splice isoforms of fibronectin that contain the extra-domain A (EDA) have been implicated to promote tumorigenesis, metastasis and vasculogenesis^6^,^9^,^12^. EDA of Fibronectin has been reported to impart the EMT phenotype and metastatic capacity to cells by interacting with α9β1 and α4β1 integrins through its C-C loop. The accessibility and abundance of EDA in majority of the tumors and its absence in most normal tissues has made EDA a useful target for therapeutic intervention that has led to the development of IST-9 antibody that selectively blocks EDA accessibility to integrins and thus prevents metastasis. Advancement of strategies utilizing EDA has lead to their use in Antibody-drug Combinations (ADC) which utilizes antibody-mediated targeted delivery of bioactive agents (e.g., Cytotoxic drugs) to tumor microenvironment, however, the efficacy of such procedures is very low owing to partial digestion of ADC’s which cause unloading of targeted drugs thus leading to undesirable effects besides the associated hypersensitivities of antibodies treatment which limit their use^19^. An alternative strategy to target the cancer cell could be achieved by targeting the cancer microenvironment, which in this study has been reached up by exploiting drugs that directly bind to EDA, limit tumor growth and thus may avoid the use of antibodies.

In this context we carried virtual screening of naturally occurring bioactive compounds from the medicinal chemiome of western Himalayan region using Molecular Docking Simulations (MDS) to figure out compounds having enhanced binding affinity towards EDA of Fibronectin. Using Molecular Docking Simulations, we screened 120 native western himalayan medicinal compounds that showed affinity with EDA domain of Fibronectin. In the initial screening, we were able to scale down to 4 compounds viz *Irigenin, Tectorigenin, Emodin* and *Safranal* based upon toxicity, RO5 and ADME parameters which determine drug-likeliness of compounds. All these compounds have been reported to prevent inflammation associated with tumors; however, a direct evidence of their role in cancer prevention is still vague^20^,^21^,^22^,^23^. Of these 4 compounds, *Irigenin*, was most effective in controlling tumor cell growth and showed maximum binding affinity with EDA as evaluated by molecular docking simulation. However, it was still not clear whether *Irigenin* possesses anti-cancer activities or has only cytotoxic behavior. To address this, we tried to evaluate the effect of *Irigenin* on tumor metastasis in human lung cancer cell lines, i.e. A549 and NCI-H522. *Irigenin* effectively diminished migratory capabilities of these cells in an *in vitro* setup pointing towards its possible role in overcoming cancer metastasis. To further corroborate these findings, we evaluated expression of metastatic markers *E-cadherin, Vimentin, Snail* and *Zeb-1* in these cells upon *Irigenin* treatment. Interestingly, *Irigenin* caused a dose-dependent inhibition of EMT, however, the same effect could not be observed in T47D cell line which does not express EDA. Therefore, it could be argued that *Irigenin* exerts its anti-metastatic effects via EDA. To further confirm these finding, we used IST-9 antibody to block EDA in A549 cells. Intriguingly, treatment of A549 cells with EDA neutralizing antibody (IST-9) attenuated their migration and invasion, thus demonstrating the similarity in mechanism of action of *Irigenin* and IST-9 antibody. Furthermore the specificity of *Irigenin* in targeting EDA was confirmed by selective expression of EDA in T47D cells which not only enhanced their metastatic capacity but also rendered *Irigenin*-responsive phenotype in them. The accentuated metastatic properties of EDA-T47D cells was however, neutralized by treatment with *Irigenin* or IST-9. These results were further corroborated by A549 EDA knockdown cells which resulted in non-responsive behaviour of these cells to either *Irigenin* or IST-9 treatment.

IST-9 antibody recognizes PEDGIHELFP epitope in the C-C loop of EDA and thus blocks its access to α9β1 and α4β1 integrins which in turn prevent EMT activation and hence metastasis. In order to understand if *Irigenin* blocks EDA in the similar fashion, we performed MM-PBSA calculations on 1001 *Irigenin*-EDA MD simulated complexes. The prominently engaging amino acids included LEU46, PHE47, PRO48, GLU58, LEU59 and GLN60 in the optimal binding site of EDA, as revealed by energy decomposition per residue diagram of EDA –*Irigenin* complex which fall in the C-C loop of EDA, the target site of commercially available IST9 antibody.

In conclusion the above results indicate that the EDA induced cell migration and invasion of lung carcinoma cells is ablated by the treatment with *Irigenin*. The current finding suggests that *Irigenin* binds to the C-C loop of EDA, thereby blocking its interaction with integrins on the cell surface and thus abrogating subsequent Epithelial-Mesenchymal transition. We envisage that this inhibition of the EDA-integrin interactions may occur either directly due to the binding of *Irigenin* to the C-C loop, thus blocking its interactions with integrins or it may create steric hindrances for binding of integrins to other sequence within EDA. In summary this study presents *Irigenin* as a lead compound, which overcomes Fibronectin EDA induced proliferation, migration and invasion of lung carcinoma cells.

## Methods

### Data Preparation

A variety of commonly used ethno-medicinal plants from the western Himalayan region were nominated and the major 120 lead bioactive compounds derived from them were used for generation of native natural product library. For structure based drug designing the Extra Domain A of the human Fibronectin protein (PDB: 1J8K) was used^24^.

### Molecular Docking Simulation

All the compounds were analyzed using AutoDock 4.2^25^ to confirm the binding mode with the C-C loop of EDA. The docking energy was obtained from the summation of van der Walls energy and hydrogen bonding energy, while as binding energy was built up from van der Walls energy and desolvation energy. Lamarckian Genetic Algorithm (GA) was considered for the run and for each ligand 10 GA runs, with 27,000 maximum generations, 0.02 rate of gene mutation and 0.8 as rate of crossover were set. A grid of 60 × 60 × 60 points in x, y, and z direction was built centered around C-C loop. Cutoff of −8 kcal/mole was set to limit the search for the lead compound against the EDA.

### Drug-likeness Prediction

The shortlisted compounds from molecular docking simulations were further screened in 3 tiers of Toxicity, RO5 and absorption distribution metabolism excretion (ADME). For toxicity, *in-silico* approaches which predict the mutagenicity and carcinogenicity were used and only those compounds were further subjected to RO5 and ADME analysis which were non-mutagenic and non-carcinogenic in nature. All the predictions were calculated using Pre-ADMET tool.

### Molecular Dynamics Simulation and MM-PBSA calculations

The Molecular Dynamics simulations (MDS) were carried out for the *Irigenin*-EDA complex using GROMACS 4.6 platform^26^ under GROMOS 43a1 force field. The force field parameter for the ligand were obtained using PRODRG server^27^. Prior to MD simulations, *Irigenin*-EDA complex was subjected to energy minimization process and its position restraint was performed in NVT and NPT ensembles at 300 k for 1 ns. The coupling scheme of Berendsen was employed in both NVT and NPT ensembles. The Particlemesh Ewald method for long-range electrostatics, a 14 Å cutoff for van der Waals interactions, a 12 Å cutoff for Coulomb interaction with updates every 2 steps, and the SHAKE algorithm to constrain bond lengths were used^28^. The trajectory generated from the simulation of the complex was studied for stability and Molecular Mechanics- Poisson Bolzmann Surface Area (MM-PBSA) calculations.

g_mmpbsa tool developed for Gromacs^29^,^30^ was used to study the binding free energy of *Irigenin*-EDA complex. The calculations were performed on r 1001 snap shots of the complex and each snap shot was taken at 100 pico seconds (ps). The binding free energy (ΔG_bind_) composed of following species:


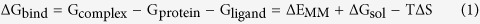



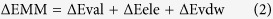



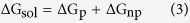



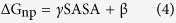


where Gcomplex, Gprotein and Gligand represent the free energy of respective species, and ΔEMM, ΔGsol, TΔS represent gas phase energy, solvation energy and entropy respectively. The gas phase energy is the summation of internal energy of bonds, angle and torsion (ΔEval), electrostatic interaction energy (ΔEele), and van der Waals interaction energy (ΔEvdw). The solvation free energy comprises of polar solvation free energy (ΔGp), and the nonpolar salvation free energy (ΔGnp). ΔGnp is the nonpolar solvation contribution, calculated with constants 0.0072 kcal mol-1 Å-2 for surface tension proportionality constant γ and 0.92 kcal mol-1 Å-2 for the nonpolar free energy for a point solute β.

### Molecular visualization & MD analysis

All the visualizations were carried out using Pymol^31^, Ligplus^32^ and Discovery Studio^33^. Graphs were plotted using Grace Program^34^ and Gnuplot^35^. The trajectories were analyzed using the inbuilt tool in the GROMACS distribution.

### Reagents and antibodies

*Irigenin* and *Tectorigenin* used in this study were isolated from the rhizomes of *Iris kashmiriana*, as reported previously^36^. *Emodin, Safranal*, 3-(4,5-dimethylthiazol-2-yl)-2,5-diphenyl-tetrazolium bromide (MTT) and dimethyl sulfoxide (DMSO) were purchased from Sigma–Aldrich Co. (St. Louis, MO, USA). DMEM, Fetal Bovine Serum (FBS), Penicillin and Streptomycin were obtained from Life Technologies Inc. (Gibco, USA). Epithelial-Mesenchymal Transition (EMT) antibody sampler kit was purchased from Cell Signaling Technology (Beverly, MA). Monoclonal antibodies against human Fibronectin EDA (IST-9) and β-actin were procured from Abcam and Sigma–Aldrich Co respectively.

### Cell culture and treatments

Human lung adenocarcinoma cell line, A549 was kindly provided by Hybridoma Lab., National Institute of Immunology (NII), New Delhi, India. Human lung adenocarcinoma cell line, NCI-H522, Human breast ductal carcinoma T47D, human cervical carcinoma Hela, and human neuroblastoma IMR-32 cell lines were procured from National Centre for Cell Science (NCCS), Pune, India. All the cell lines were cultured in DMEM (Gibco) supplemented with 10% fetal bovine serum (Gibco) and 1% penicillin-streptomycin (Invitrogen) under a humidified 5% CO_2_ atmosphere at 37 °C in an incubator. All the compounds used in the study were prepared in DMSO and delivered to cell culture in complete medium. The EDA neutralizing antibody (IST-9) was directly added to the cultures at optimal effective concentration of 20 μg/ml^12^.

### Cell proliferation assay

Cells were seeded in a 24-well plate, allowed to grow overnight in 10% FBS supplemented DMEM. After 24 h of seeding, different concentration of compounds (*Irigenin, Tectorigenin, Curcumin and Safranal*) dissolved in DMSO were added. Controls consisted of cells treated with DMSO (vehicle control). Each concentration was tested in triplicate. The cells were incubated at 37 °C in a humidified incubator with 5% CO_2_ for 24 h. After 24 h, the media was removed and MTT solution was added to the cells at a concentration of 0.1 mg/ml followed by incubation for 4 h at 37 °C in dark. Then the supernatant was removed and an equal volume of DMSO was added to dissolve the formazan crystals. The absorbance was measured at 565 nm against background absorption at 650 nm in a Microplate Reader (Bio-Tek Instruments, USA).

### Wound healing assay

Cells were grown to confluency in 6 well plates. The culture media was then removed and the confluent monolayer was scratched with a 200 μl pipette tip, washed twice with PBS to remove detached cells and photographed (T_0_). Cells were then treated with 20 μg/ml of EDA neutralizing antibody (IST-9) or indicated concentrations of *Irigenin* or DMSO (control) for 24 h. Finally the wounds were observed using an inverted microscope and photographed (T_24_). The assay was repeated 3 independent times. The extent of wound closure was calculated as the percentage of the initial wound until final wound closure at 24 h time point using ImageJ software.

### Invasion assay

The invasion assay was performed in polycarbonate filter inserts (8 mm pore size, Corning), precoated with Matrigel. Briefly, cells suspended in 500 μl of DMEM were seeded onto membranes of the transwell inserts to which 20 μg/ml IST-9 or indicated concentrations of *Irigenin* were added. These inserts were placed into the wells of 24-well plate which served as lower chambers and contained DMEM supplemented with 10% FBS as a chemotactic agent. After 24 h incubation, the un-migrated cells in the upper chamber of the filters were removed by wiping the top of the insert membranes with a cotton swab. The cells that migrated and adhered to the other side of the filter were fixed in 3.7% formaldehyde for 20 min, stained with crystal violet and counted per five fields. The experiment was repeated at least three independent experiments.

### Construction of pcDNA-EDA construct and transient transfection

The following primers were designed to amplify the EDA exon region of the human Fibronectin gene. Forward primer 5–GAATTCAGTCAGCCTCTGGTTCAGAC–3 Reverse primer 5–GGATCCCTTCAGGTCAGTTGGTGCAG–3 using RNA isolated from A549 cells. The 310 base pair EDA PCR product was separated on 1.5% agarose gel followed by its gel extraction and purification using Qiagen columns. Finally the purified product was ligated into the pcDNA 3.1 expression vector (Addgene) using BAMHI and ECORI restriction sites. The pcDNA-EDA construct was transfected into T47D cells with Polyethylenimine (Polysciences) when cells became 70–75% confluent. Cells were transfected with 0.5, 1 or 2 μg of the pcDNA-EDA construct; however transfection with 2 μg of the construct proved to be highly efficient as indicated by semi-quantitative reverse transcription-PCR and western blotting.

### shRNA transfection

Fibronectin shRNA Plasmid (Santa Cruz Biotechnology) a pool of 3 target-specific lentiviral vector plasmids each encoding 19–25 nt (plus hairpin) shRNAs designed to knock down gene expression of EDA were used to transfect A549 cells. Control consisted of shRNA plasmid encoding a scrambled shRNA sequence that does not lead to the specific degradation of any known cellular mRNA. Briefly for each transfection, 1 μg shRNA plasmid was used and transfections were carried using shRNA transfection reagent as per manufacturer’s protocol. 48 hours post-transfection, cells were subjected to puromycin treatment at 2 μg/ml to select for stable transfectants. Selection continued until the untransfected cells were killed. The cells were then tested for EDA knockdown by evaluating the expression levels of EDA mRNA and protein.

### Semi-quantitative Reverse Transcription-PCR

Expression of EDA in T47D cells after transfection with pcDNA-EDA construct and silencing in A549 cells was assessed by performing reverse transcription-PCR using cDNA prepared from total RNA of the cells. Primers used were: Forward primer 5–AGTCAGCCTCTGGTTCAGAC–3 and reverse primer 5–CTTCAGGTCAGTTGGTGCAG–3. Tubulin was used as internal control.

### Western blotting

The procedure was performed as described previously^23^. Briefly IST-9 or *Irigenin* treated A549 cells were harvested, lysed in RIPA buffer and the protein concentrations were determined by BCA method (Thermo Pierce). Equal amounts of protein from whole-cell lysates were separated on SDS-polyacrylamide gels (10–12%) and then electrophoretically transferred onto PVDF membranes. Blots were incubated with primary antibodies against Fibronectin (1:3000), EDA (1:1000), E-cadherin (1:1000), Vimentin (1:1000), Zeb-1 (1:2000), Snail (1:2000) and β-actin (1:500) at 4 °C overnight. After washings, the blots were incubated with appropriate IR-tagged secondary antibodies (1:10,000) for 1 h at room temperature. The blots were then scanned in an infrared image scanner (Licor Biosciences).

### Statistical analysis

Statistical analysis was carried out using One-Way Analysis Of Variance (ANOVA), and the level of significance was tested at p value ranging from 0.01 to 0.001. Data are expressed as the mean ± SD of at least three independent experiments.

## Additional Information

**How to cite this article**: Amin, A. *et al. Irigenin*, a novel lead from Western Himalayan chemiome inhibits Fibronectin-Extra Domain A induced metastasis in Lung cancer cells. *Sci. Rep.* **6**, 37151; doi: 10.1038/srep37151 (2016).

**Publisher’s note**: Springer Nature remains neutral with regard to jurisdictional claims in published maps and institutional affiliations.

## Supplementary Material

Supplementary Information

Supplementary Information

Supplementary Information

## References

[b1] Han S., Khuri F. R. & Roman J. Fibronectin stimulates non–small cell lung carcinoma cell growth through activation of Akt/mammalian target of rapamycin/S6 kinase and inactivation of LKB1/AMP-activated protein kinase signal pathways. Cancer research 66, 315–323, doi: 10.1158/0008-5472.can-05-2367 (2006). 16397245

[b2] Harima A. . Fibronectin promotes cell proliferation of human pre-B cell line via its interactions with VLA-4 and VLA-5. Hematology 13, 236–243, doi: 10.1179/102453308x348315 (2008). 18796250

[b3] Kornblihtt A. . The fibronectin gene as a model for splicing and transcription studies. The FASEB Journal 10, 248–257 (1996). 8641558 10.1096/fasebj.10.2.8641558

[b4] White E. S., Baralle F. & Muro A. New insights into form and function of fibronectin splice variants. The Journal of pathology 216, 1–14, doi: 10.1002/path.2388 (2008). 18680111 PMC4630009

[b5] Muro A. F. . Regulated splicing of the fibronectin EDA exon is essential for proper skin wound healing and normal lifespan. The Journal of cell biology 162, 149–160, doi: 10.1083/jcb.200212079 (2003). 12847088 PMC2172721

[b6] Xiang L. . The extra domain A of fibronectin increases VEGF-C expression in colorectal carcinoma involving the PI3K/AKT signaling pathway. Plos ONE 7, e35378, doi: 10.1371/journal.pone.0035378 (2012). 22496919 PMC3322170

[b7] Ou J. . Fibronectin extra domain A (EDA) sustains CD133+/CD44+ subpopulation of colorectal cancer cells. Stem cell research 11, 820–833, doi: 10.1016/j.scr.2013.05.009 (2013). 23811539 PMC3917514

[b8] Ou J.-J., Wu F. & Liang H.-J. Colorectal tumor derived fibronectin alternatively spliced EDA domain exserts lymphangiogenic effect on human lymphatic endothelial cells. Cancer biology & therapy 9, 186–191, doi: 10.4161/cbt.9.3.10651 (2010). 20023414

[b9] Rybak J. N., Roesli C., Kaspar M., Villa A. & Neri D. The Extra-domain A of Fibronectin Is a Vascular Marker of Solid Tumors and Metastases. Cancer Research 67, 10948–10957, doi: 10.1158/0008-5472.can-07-1436 (2007). 18006840

[b10] Liao Y.-F., Gotwals P. J., Koteliansky V. E., Sheppard D. & Van De Water L. The EIIIA Segment of Fibronectin Is a Ligand for Integrins α9β1 and α4β1Providing a Novel Mechanism for Regulating Cell Adhesion by Alternative Splicing. Journal of Biological Chemistry 277, 14467–14474, doi: 10.1074/jbc.m201100200 (2002). 11839764

[b11] Shinde A. V. . Identification of the peptide sequences within the EIIIA (EDA) segment of fibronectin that mediate integrin α9β1-dependent cellular activities. Journal of Biological Chemistry 283, 2858–2870, doi: 10.1074/jbc.m708306200 (2008). 17967897

[b12] Ou J. . Endothelial cell-derived fibronectin extra domain A promotes colorectal cancer metastasis via inducing epithelial–mesenchymal transition. Carcinogenesis, doi: 10.1093/carcin/bgu090 (2014). 24743511

[b13] Villa A. . A high‐affinity human monoclonal antibody specific to the alternatively spliced EDA domain of fibronectin efficiently targets tumor neo‐vasculature *in vivo*. International journal of cancer 122, 2405–2413, doi: 10.1002/ijc.23408 (2008). 18271006

[b14] Wieckowski S. . Therapeutic efficacy of the F8-IL2 immunocytokine in a metastatic mouse model of lung adenocarcinoma. Lung Cancer 88, 9–15, doi: 10.1016/j.lungcan.2015.01.019 (2015). 25682318

[b15] Chikan N. A., Bhavaniprasad V., Anbarasu K., Shabir N. & Patel T. N. From natural products to drugs for epimutation computer-aided drug design. Applied biochemistry and biotechnology 170, 164–175, doi: 10.1007/s12010-013-0158-6 (2013). 23483409

[b16] Malik A. H., Khuroo A. A., Dar G. & Khan Z. Ethnomedicinal uses of some plants in the Kashmir Himalaya. Indian Journal of Traditional Knowledge 10, 362–366 (2011).

[b17] Lipinski C. A. Lead- and drug-like compounds: the rule-of-five revolution. Drug discovery today. Technologies 1, 337–341, doi: 10.1016/j.ddtec.2004.11.007 (2004). 24981612

[b18] Moroy G., Martiny V. Y., Vayer P., Villoutreix B. O. & Miteva M. A. Toward in silico structure-based ADMET prediction in drug discovery. Drug discovery today 17, 44–55, doi: 10.1016/j.drudis.2011.10.023 (2012). 22056716

[b19] Baldo B. Adverse events to monoclonal antibodies used for cancer therapy: Focus on hypersensitivity responses. OncoImmunology, 2, e26333, doi: 10.4161/onci.26333 (2013). 24251081 PMC3827071

[b20] Malaekeh-Nikouei B., Mousavi S. H., Shahsavand S., Mehri S., Nassirli H. & Moallem S. A. Assessment of Cytotoxic Properties of *Safranal* and Nanoliposomal *Safranal* in Various Cancer Cell Lines. Phytotherapy Research 27, 1868–1873, doi: 10.1002/ptr.4945 (2013). 23494763

[b21] Zhu T., Zhang W., Feng S. & Yu H. *Emodin* suppresses LPS-induced inflammation in RAW264.7 cells through a PPARγ-dependent pathway. International Immunopharmacology 34, 16–24, doi: 10.1016/j.intimp.2016.02.014 (2016). 26910236

[b22] Ahn K. S. . Inhibitory effects of *Irigenin* from the rhizomes of Belamcanda chinensis on nitric oxide and prostaglandin E2 production in murine macrophage RAW 264.7 cells. Life Sciences 78, 2336–2342, doi: 10.1016/j.lfs.2005.09.041 (2006). 16307761

[b23] Amin A. . *Tectorigenin* ablates the inflammation-induced epithelial–mesenchymal transition in a co-culture model of human lung carcinoma. Pharmacological Reports 67, 382–387, doi: 10.1016/j.pharep.2014.10.020 (2015). 25712668

[b24] Niimi T. . NMR structure of human fibronectin EDA. Journal of biomolecular NMR 21, 281–284, doi: 10.1023/a:1012947209393 (2001). 11775745

[b25] Morris G. M. . AutoDock4 and AutoDockTools4: Automated docking with selective receptor flexibility. Journal of computational chemistry 30, 2785–2791, doi: 10.1002/jcc.21256 (2009). 19399780 PMC2760638

[b26] Hess B., Kutzner C., Van Der Spoel D. & Lindahl E. GROMACS 4: algorithms for highly efficient, load-balanced, and scalable molecular simulation. Journal of chemical theory and computation 4, 435–447, doi: 10.1021/ct700301q (2008). 26620784

[b27] SchuÈttelkopf A. W. & Van Aalten D. M. PRODRG: a tool for high-throughput crystallography of protein–ligand complexes. Acta Crystallographica Section D: Biological Crystallography 60, 1355–1363, doi: 10.1107/s0907444904011679 (2004). 15272157

[b28] Essmann U. . A smooth particle mesh Ewald method. The Journal of chemical physics 103, 8577–8593, doi: 10.1063/1.470117 (1995).

[b29] Baker N. A., Sept D., Joseph S., Holst M. J. & McCammon J. A. Electrostatics of nanosystems: application to microtubules and the ribosome. Proceedings of the National Academy of Sciences 98, 10037–10041 (2001). 10.1073/pnas.181342398 PMC56910 11517324

[b30] Kumari R., Kumar R. & Lynn A. g_mmpbsa — A GROMACS Tool for High-Throughput MM-PBSA Calculations. Journal of chemical information and modeling 54, 1951–1962, doi: 10.1021/ci500020m (2014). 24850022

[b31] DeLano W. L. The PyMOL Molecular Graphics System, Version 1.8 Schrödinger, LLC, Cambridge, MA. https://www.pymol.org (2002).

[b32] Laskowski R. A. & Swindells M. B. LigPlot+: multiple ligand–protein interaction diagrams for drug discovery. Journal of chemical information and modeling 51, 2778–2786, doi: 10.1021/ci200227u (2011). 21919503

[b33] Accelrys Software Inc. Discovery Studio Modeling Environment, Version 3.5, San Diego, CA, USA. http://www.accelrys.com (2012).

[b34] Turner, P. J. XMGRACE, Version 5.1. 19. Center for Coastal and Land-Margin Research, Oregon Graduate Institute of Science and Technology, Beaverton, OR, USA. http://plasma-gate.weizmann.ac.il/Grace/ (2005).

[b35] Williams T. & Kelley C. Gnuplot 4.4: an interactive plotting program. http://www.gnuplot.info/ (2011).

[b36] Amin A. . Investigating the pharmacological potential of Iris kashmiriana in limiting growth of epithelial tumors. Pharmacognosy Journal 5, 170–175, doi: 10.1016/j.phcgj.2013.07.003 (2013).

