# Supplementary material for: Irigenin, a novel lead from Western Himalayan chemiome inhibits Fibronectin-Extra Domain A induced metastasis in Lung cancer cells
Source: Sci Rep. 2016 Nov 16;6:37151. doi: 10.1038/srep37151 (PMC5111083; doi:10.1038/srep37151)
Supplement: Supplementary Information [file srep37151-s1.pdf]

***Irigenin*, a novel lead from Western Himalayan chemiome inhibits Fibronectin-Extra Domain A induced metastasis in Lung cancer cells.**

Asif Amin[a‡], Naveed Anjum Chikan[a,b,‡], Taseem A. Mokhdomi[a], Shoiab Bukhari[a,c], Aabid Koul[a], Basit Shah[a], Fatemeh Rahimi[d] Asrar H. Wafai[a], Ayub Qadri[d] and Raies A. Qadri[a]*

Supplementary Data

Figure 6a

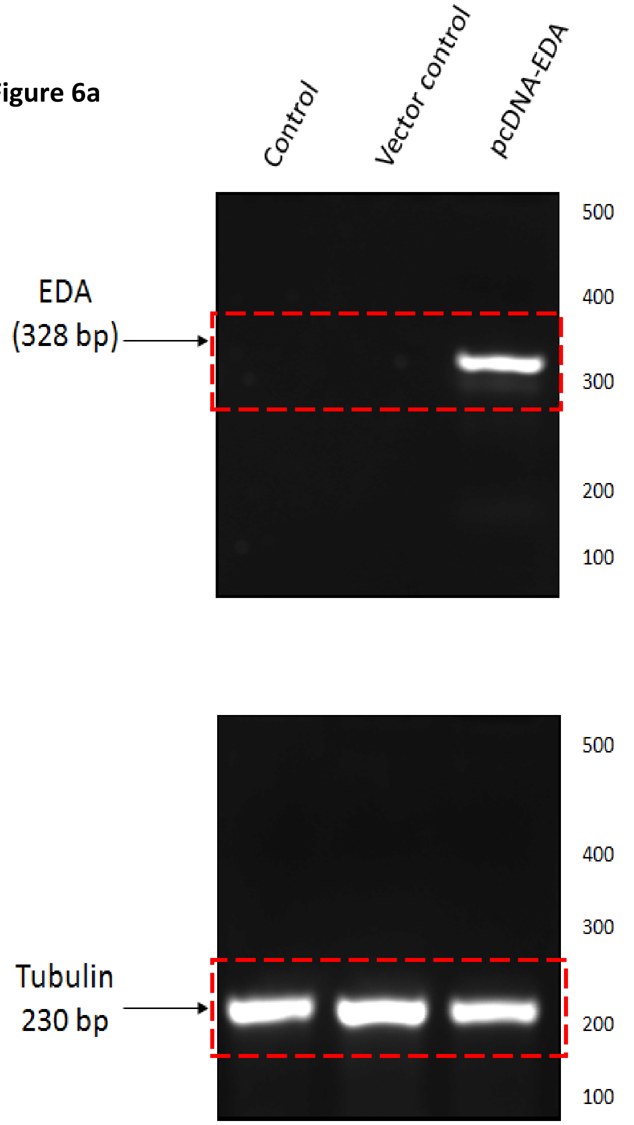

Figure 6b

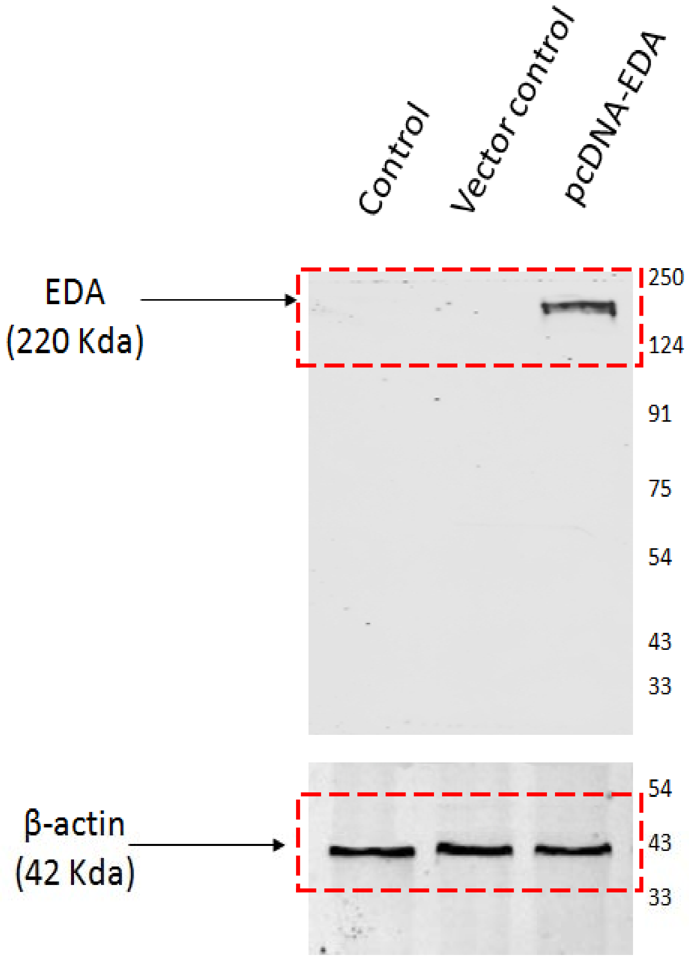

Figure 6c

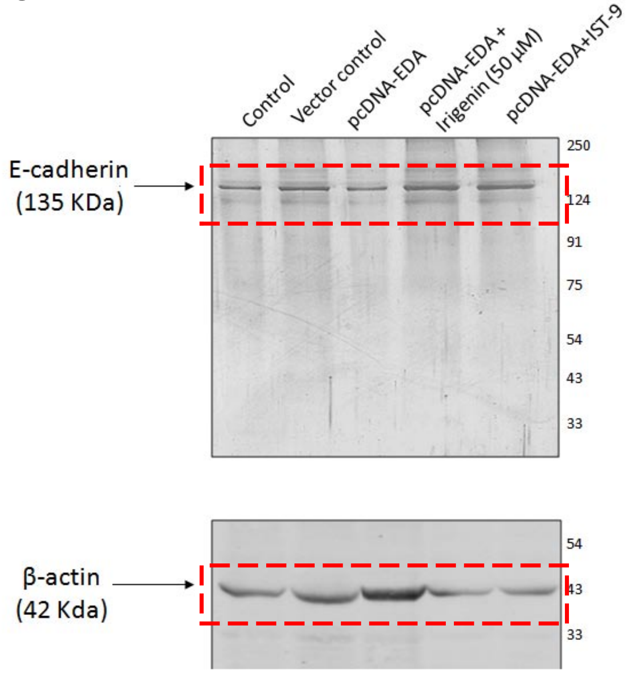

Figure 6d

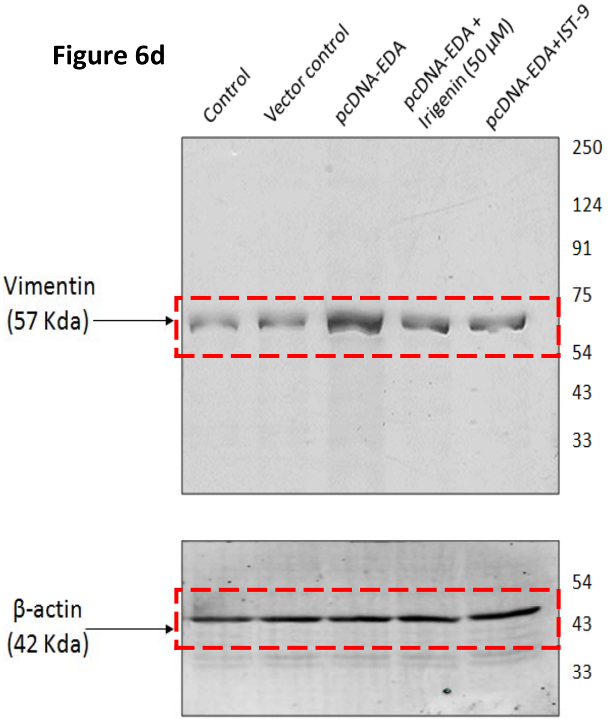

Figure 6e

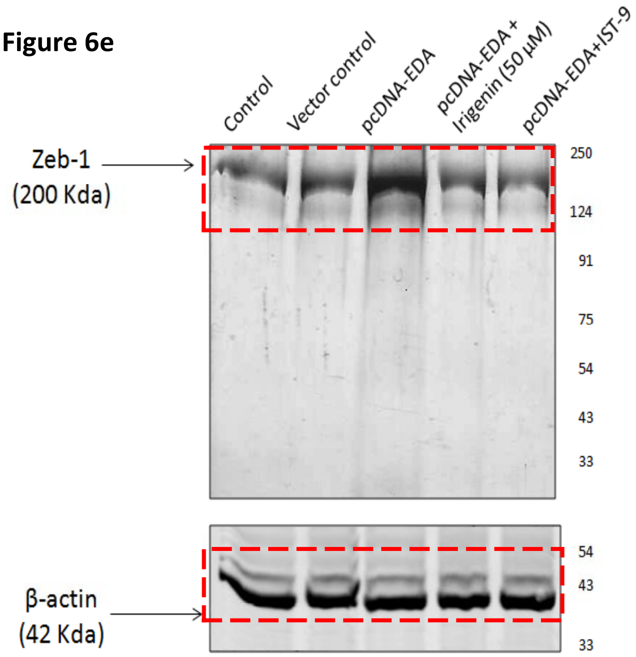

Figure 6f

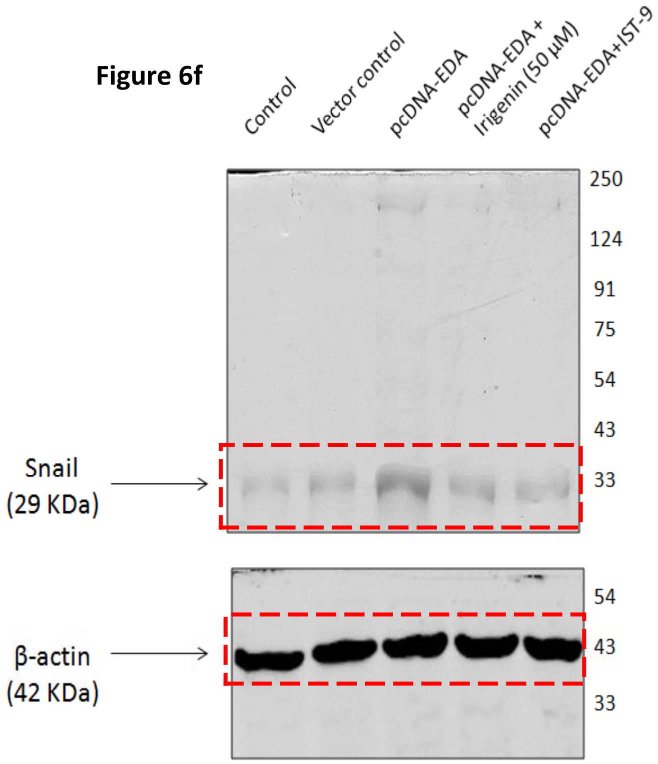

Supplementary Figure S9: Uncropped Immunoblots of Figure 6 depicting cropped area (red line)

Figure 7a

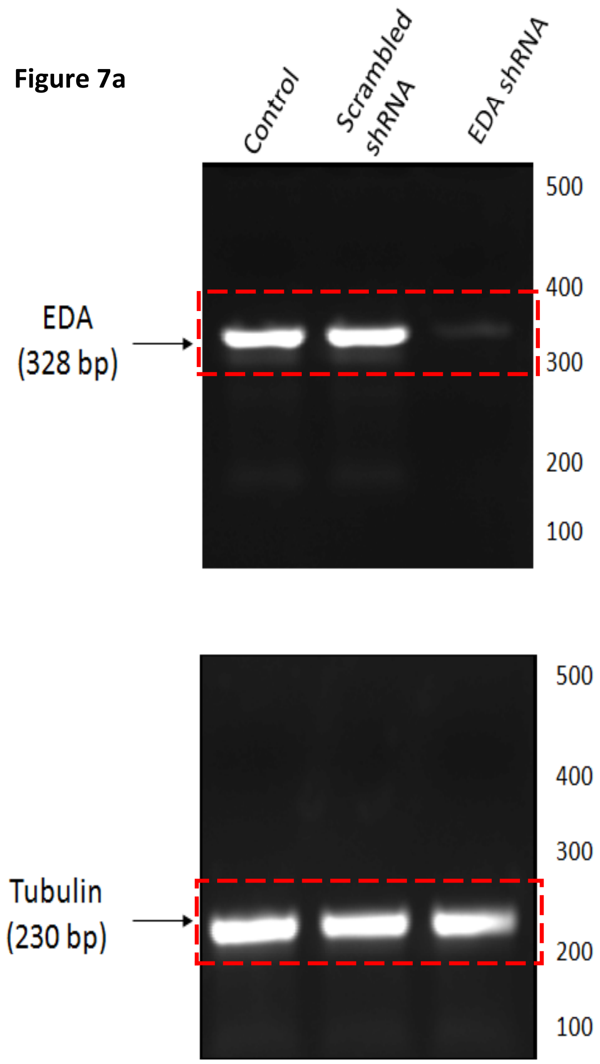

Figure 7b

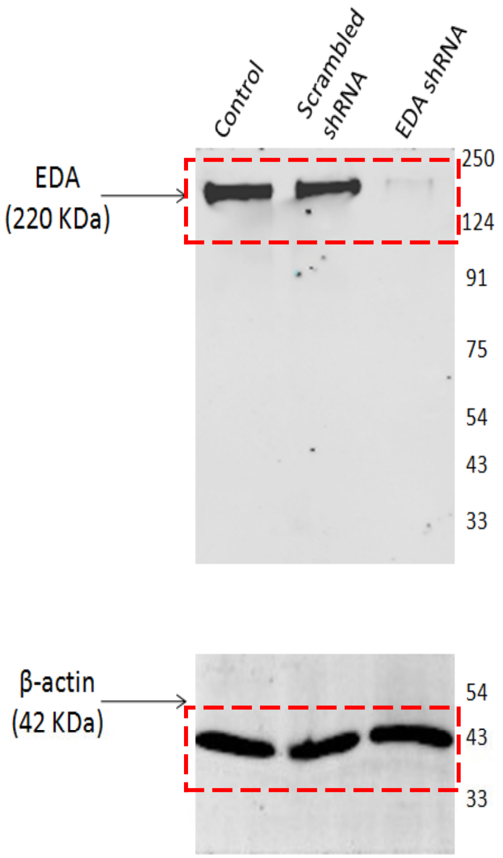

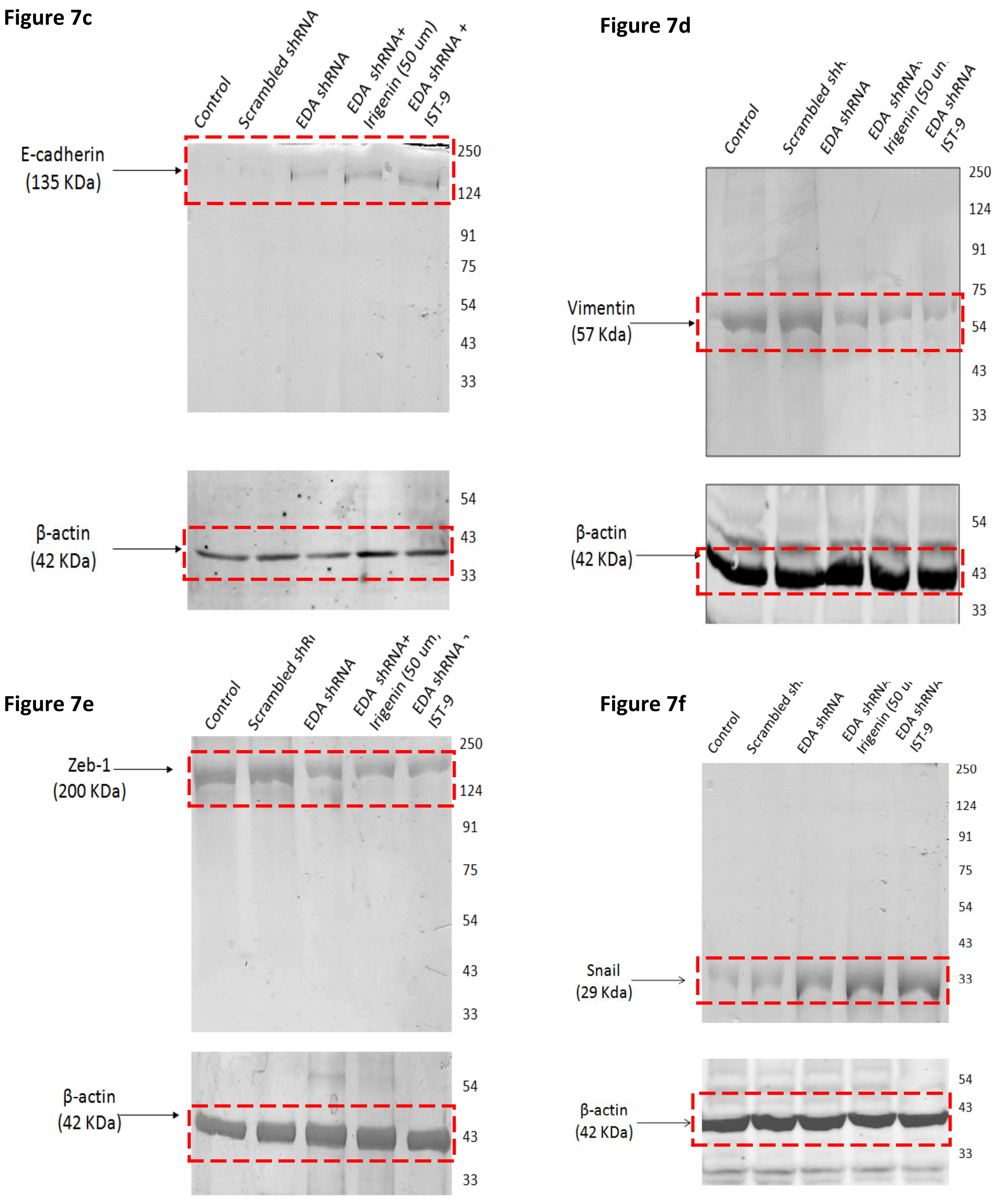

Supplementary Figure S10: Uncropped Immunoblots of Figure 7 depicting cropped area (red line)
