# Supplementary material for: Irigenin, a novel lead from Western Himalayan chemiome inhibits Fibronectin-Extra Domain A induced metastasis in Lung cancer cells
Source: Sci Rep. 2016 Nov 16;6:37151. doi: 10.1038/srep37151 (PMC5111083; doi:10.1038/srep37151)
Supplement: Supplementary Information [file srep37151-s2.pdf]

Irigenin, a novel lead from Western Himalayan chemiome inhibits Fibronectin-Extra Domain A induced metastasis in Lung cancer cells.

Asif Amin[a‡], Naveed Anjum Chikan[a,b,‡], Taseem A. Mokhdomi[a], Shoiab Bukhari[a,c], Aabid [a], Basit Shah[a], Fatemeh Rahimi[d] Asrar H. Wafai[a], Ayub Qadri[d] and Raies A. Qadri[a]*

Supplementary Data

TableS1: Library of bioactive compounds obtained from commonly used medicinal compounds of western Himalayan region.

**Drug**	**Plant source**	**CID No.**	**CSID No.**
Absinthin	*Artemisia absinthium Linn*	CID 442138	
Aescin	*Aesculus indica colebr. & Camb. (Hippocastanaceae)*		CSID 23089563
Aesculin	*Aesculus hippocastanum Linn*	CID 5281417	
Aglycone	*Eryngium coeruleum Bieb.*		CSID 16736194
Alantolactone	*Inula racemosa HK. F.*	CID 72724	
Amaroswerin	*Gentiana kurroo Royle*	CID 45359883	
Andromedotoxin (Acetyllandromedol)	*Rhododendron campanulatum D. Don.*		CSID 7827535
Apigenin	*Meconopsis horridula*	CID 5280443	
Apigravin	*Apium graveolens L.*		CSID 30776837
Apiumoside (Apiin)	*Apium graveolens L.*		CSID 4444321
Arnidiol	*Calendula officinalis Linn.*	CID 470259	
Artabsin	*Artemisia absinthium L*	CID 442146	
Artemisinin	*Artemisia drancunculus L.*	CID 68827	
Asarone	*Acorus calamus Linn*		CSID 552532
Ascaridol	*Chenopodium ambrosioides L.*	CID 10545	

Astragalin	*Aesculus indica colebr. & Camb. (Hippocastanaceae)*	CID 5282102	
Atisine	*Aconitum heterophyllum Wallich ex Royle*	CID 9548630	
Atropine	*Atropa acuminata*	CID 174174	
Avicularin	*Polygonum aviculare Linn.*	CID 5490064	
Azulene	*Achillea millefolium L.*	CID 9231	
Barrigenol A1	*Eryngium coeruleum Bieb.*	CID 177603	
Barringenol R1	*Eryngium coeruleum Bieb.*	CID 44202129	
β-Dihydrofucosterol (Azuprostat)	*Euphorbia helioscopia Linn.*	CID 457801	
Berberine	*Berberis aristata DC*	CID 2353	
Bergapten	*Apium graveolens L.*	CID 2355	
Bergenin	*Bergenia stracheyi Hook*	CID 2356	
Bikhaconitine	*Aconitum violaceum Jacq.*	CID 441713	
Borneol	*Prangos pabularia Lindl.*	CID 64685	
Camphene	*Prangos pabularia Lindl.*	CID 6616	
Cannabinin	*Cannabis sativus Linn.*		CSID 8372337
Cannabinol	*Cannabis sativus Linn.*	CID 2543	
Capillarin	*Artemisia drancunculus L.*		CSID 2340963
Carpesterol	*Solanum xanthocarpum*	CID 21155918	
Carvacrol	*Carum carvi Linn.*	CID 10364	
Carvone	*Carum carvi Linn.*		CSID 21106424
Celerin	*Apium graveolens L.*		CSID 137753

Choline	*Dictamnus albus Linn.*	CID 305	
Chrysophanic Acid (Chrysophanol)	*Rheum emodi Wall.*	CID 10208	
Citronellol	*Mentha arvensis Linn.*	CID 8842	
Colchicine	*Colchicum leteum Baker*	CID 6167	
Convolvulin (Convolvin)	*Convolvulus arvensis L.*		CSID 245689
Coriandrol	*Coriandum sativum Linn.*	CID 67179	
Coumarin	*Angelica glauca Edgew.*	CID 323	
Cryptopine	*Fumaria indica L.*	CID 72616	
Cyanidin	*Asparagus racemosus Willd.*	CID 68247	
Diosgenin	*Dioscorea deltoidea Wall*	CID 99474	
Ecdysterone	*Achyranthes aspera L.*	CID 5459840	
Emodin	*Rheum emodii*	CID 3220	
Ephedrine	*Ephedra gerardiana*	CID 5032	
Esculetin	*Koelpinia linearis Pall.*	CID 5281416	
Etoposide	*Podophyllum hexandrum Royle*	CID 36462	
Faradiel	*Calendula officinalis Linn.*	CID 122856	
Filicin	*Dryopteris filixmas L.*	CID 197044	
Fumaramine	*Fumaria indica L.*	CID 6450006	
Gentianine	*Gentiana kurroo Royle*	CID 354616	
Gentiopicrin	*Gentiana kurroo Royle*		CSID 32697064
Harmaline	*Peganum harmala Linn.*	CID 5280951	

Harmalol	*Peganum harmala Linn.*	CID 5353656	
Harmine	*Peganum harmala Linn.*	CID 5280953	
Hetisine	*Aconitum heterophyllum Wallich ex Royle*		CSID 10226875
Hetisinone	*Aconitum heterophyllum Wallich ex Royle*		CSID 10226887
Hexacosane	*Anagallis arvensis L.*		CSID 11901
Hyoscine	*Datura stramonium Linn*	CID 3000322	
Hyoscyamine	*Datura stramonium Linn*	CID 64692	
Hyperoside	*Asparagus racemosus Willd.*	CID 5281643	
Imperialine (Kashmirine)	*Fritillaria imperialis Linn.*	CID 442977	
Indaconitine	*Aconitum violaceum Jacq.*	CID 441740	
Inokosterone	*Achyranthes aspera L.*	CID 441828	
Intybin	*Cichorium intybus L.*	CID 174863	
Irigenin	*Iris kashmiriana*	CID 5464170	
Isoalantolactone	*Inula racemosa HK. F.*	CID 73285	
Isoatisine	*Aconitum heterophyllum Wallich ex Royle*	CID 245006	
Isoimperatorin	*Anthriscus nemorosa Spreng*	CID 68081	
Isopimpinellin	*Apium graveolens L.*	CID 68079	
Kaempferol	*Anagallis arvensis L.*	CID 5280863	
Lactucin	*Cichorium intybus L.*	CID 3756497	
Lactucopicrin	*Lactuca serriola Linn.*		CSID 2723771
Laureline	*Skimmia laureola Hk. f.*	CID 821373	

Lignans	*Daphne oleoides*	CID 9917980	
Luteolin	*Meconopsis horridula*	CID 5280445	
Malvalic Acid	*Althaea officinalis L.*	CID 10416	
Marrubin	*Marrubium vulgare L.*		CSID 66118
Maslinic Acid	*Epilobium angustifolium Linn.*	CID 73659	
Mezerein	*Daphne oleoides*	CID 9549167	
Myrcene	*Prangos pabularia Lindl.*	CID 31253	
Nepetalactone	*Nepeta cataria*	CID 161367	
Obaculactone (Dictamnolactone)	*Dictamnus albus Linn.*	CID 65071	
Obtusilobin (Obtusifolin)	*Anemone obtusiloba D. Don*	CID 3083575	
Oleanolic Acid	*Epilobium angustifolium Linn.*	CID 10494	
Osthenol	*Apium graveolens L.*	CID 5320318	
p-Cymene	*Thymus serpyllum Linn.*	CID 7463	
Peganine	*Peganum harmala Linn.*	CID 72610	
Pinoresinol	*Daphne oleoides*	CID 234817	
Podophyllotoxin	*Podophyllum hexandrum Royle*	CID 10607	
Prangolarin	*Anthriscus nemorosa Spreng*	CID 17536	
Protopine	*Argemone mexicana L.*	CID 4970	
Quercetin	*Aesculus indica colebr. & Camb. (Hippocastanaceae)*	CID 5280343	
Rutin	*Aesculus indica colebr. & Camb. (Hippocastanaceae)*	CID 5280805	
Sabinen	*Nepeta cataria*	CID 18818	

Safranal	*Crocus sativus L.*	CID 61041	
Sanguinarine	*Fumaria indica L.*	CID 5154	
Santonin	*Artemisia maritima Linn*	CID 221071	
scopoletin	*Artemisia drancunculus L.*	CID 5280460	
Sesamin	*Daphne oleoides*	CID 72307	
Seselin	*Apium graveolens L.*	CID 68229	
Sesquiterpene	*Acorus calamus L.*		CSID 19953446
shikonin	*Arnebia guttata Bunge*	CID 479503	
Sitosterol	*Adonis aestivalis L.*	CID 222284	
Spathulenol	*Nepeta cataria*	CID 522266	
Stigmasterol	*Asparagus racemosus Willd.*	CID 5280794	
Taraxacin	*Taraxacum officinale*	CID 5241825	
Taraxasterol	*Taraxacum officinale*	CID 5270604	
Tectoreginin	*Iris kashmiriana*	CID 5281811	
Trigonelline	*Achillea millefolium L.*	CID 5570	
Tropane	*Atropa acuminata*	CID 637986	
Umbelliferone	*Skimmia laureola Hk. f.*	CID 5281426	
Ursolic Acid	*Epilobium angustifolium Linn.*	CID 64945	
Valepotriate	*Valeriana jatamansi Jones*	CID 442436	
Xylopinine (Govanine)	*Corydalis govaniana*	CID 226520	
1-Hentriacontanol	*Aesculus indica colebr. & Camb. (Hippocastanaceae)*		CSID 61640

1,4-Cineole (Natural)	*Artemisia maritima L.*	CID 10106	
7-Methoxycoumarin (herniarin)	*Artemisia drancunculus L.*	CID 10748	
16-Hentriacontanone (palmitone)	*Aesculus indica colebr. & Camb. (Hippocastanaceae)*		CSID 85480

TableS2: Gibbs free energy score, Physico-Chemical and biological properties of bioactive compounds

**Compound**	**Gibbs Free Energy (kcal/mol)**	**Drug-likeness**						**Absorption Distribution Metabolism Excretion**				
		Mutagenicity	Carcinogenicity	HBA	HBD	TPSA	MW	BBB	Caco2	HIA	MDCK	PPB
Carpesterol	-13.3787	yes	No									
Peganine	-11.0601	Yes	No									
Cyanidin	-10.4529	Yes	No									
Isoimperatorin	-10.3158	Yes	No									
Capillarin	-10.2923	Yes	Yes									
Atropine	-10.195	No	Yes									
Taraxacin	-10.0508	Yes	Yes									
Aescin	-10.0481											
Irigenin	-10.0414	No	No	Q	Q	Q	Q	0.043263	9.21056	86.80184	1.77078	79.69777
Hyoscyamine	-10.0339	Yes	No									
Osthenol	-9.58138	Yes	No									
Sabinen	-9.50068	Yes	No									
p-cymene	-9.42523	Yes	Yes									
Sanguinarine	-9.35777	Yes	Yes									
Coumarin	-9.33665	Yes	Yes									
Safranal	-9.29266	No	No	Q	Q	Q	Q	1.06267	23.0033	100	249.139	15.17273
Cannabinol	-9.18551	No	Yes									
Myrcene	-9.10893	Yes	No									
Ephedrine	-9.01662	Yes	No									
Carvone	-8.96438	Yes	No									
Intybin	-8.92803	No	Yes									
Azulene	-8.92402	Yes	Yes									
Carvacrol	-8.89384	Yes	No									
Faradiol	-8.89069	No	Yes									

Bergenin	-8.86525	Yes	No									
Hyperoside	-8.78649	No	No	V	V	Q	Q					
Avicularin	-8.67923	Yes	No									
Taraxasterol	-8.49042	Yes	No									
Emodin	-8.43599	No	No	Q	Q	Q	Q	0.668094	20.2745	90.42972	44.9367	100
Maslinic Acid	-8.40496	No	Yes									
Kaempferol	-8.36988	Yes	No									
Asarone	-8.34362	Yes	Yes									
Ecdysterone	-8.33694	No	Yes									
Apigenin	-8.32192	Yes	No									
Obaculactone	-8.29701	Yes	No									
Umbelliferone	-8.281	Yes	Yes									
Amaroswerin	-8.23858	No	Yes									
Tectorigenin	-8.23005	No	No	Q	Q	Q	Q	0.126227	5.59415	88.18405	16.8164	87.63624
Oleanolic Acid	-8.21107	No	Yes									
Celerin	-8.15443	Yes	No									
Barringenol A1	-8.02007	No	Yes									
Obtusifolin	-8.00917	Yes	No									
Esculetin	-7.94481											
7-Methoxycoumarin	-7.94033											
Arnidiol	-7.92807											
Chrysophanic Acid	-7.90567											
Astragalin	-7.90475											
Fumaramine	-7.8825											
Ursolic Acid	-7.83721											
Sitosterol	-7.78748											

Marrubin	-7.76988											
Aesculin	-7.76358											
Shikonin	-7.70705											
Hetisine	-7.62995											
Seselin	-7.57116											
Ascaridol	-7.54342											
Hyoscine	-7.5294											
Malvalic Acid	-7.48294											
Quercetin	-7.4617											
Inokosterone	-7.41245											
Luteolin	-7.39778											
Imperialine	-7.38899											
Santonin	-7.25135											
Camphene	-7.22408											
Stigmasterol	-7.2086											
Diosgenin	-7.20553											
Sesquiterpene	-7.19878											
Isopimpinellin	-7.10912											
Absinthin	-7.08183											
Filicin	-7.06472											
Colchicine	-7.05574											
Isoatisine	-6.99133											
Laureline	-6.98638											
Lactucopicrin	-6.96841											
ß-Dihydrofucosterol	-6.95752											
Scopoletin	-6.88906											
Andromedotoxin	-6.86834											
Gentiopicrin	-6.84314											

Hetisinone	-6.83401											
Harmalol	-6.82384											
Podophyllotoxin	-6.82185											
Harmaline	-6.81269											
Govanine	-6.78949											
Bergapten	-6.75754											
Lactucin	-6.75577											
Sesamin	-6.74654											
Harmine	-6.72931											
Protopine	-6.71054											
Apiin	-6.68698											
Artabsin	-6.64119											
Berberine	-6.62831											
Valepotriate	-6.59665											
Barringenol A1	-6.59396											
Apigravin	-6.52009											
Artemisinin	-6.51894											
Spathulenol	-6.4596											
Alantolactone	-6.43428											
Isoalantolactone	-6.42729											
Convolvin	-6.33586											
Rutin	-6.23839											
Pinoresinol	-6.23388											
Citronellol	-6.21042											
Nepetalactone	-6.19349											
Atisine	-6.18617											
1,4-Cineole	-6.02481											
Tropane	-5.85134											

Borneol	-5.81539											
Prangolarin	-5.73041											
Cryptopine	-5.59433											
Coriandrol	-5.59364											
Trigonelline	-5.55835											
16-Hentriacontanone	-5.45843											
Lignans	-5.4099											
Hexacosane	-5.35611											
Cannabinin	-5.15507											
Mezerein	-4.89756											
Choline	-4.69109											
Etoposide	-4.47279											
1-Hentriacontanol	-1.20476											
Aglycone	24.0676											

Q: *Qualified;* **V**: *Violated*

Table S3: Auto Dock analysis of four compounds. The ligand binding pocket and the hydrogen bond formation was calculated using Discovery Studio 3.5 software. The bold amino acids represent the one which are involved in forming hydrogen bond with the ligand

NAME	Chem ID	ΔG Kcal/mol	Ligand binding pocket	H-bonds
Irigenin	5464170	-10.04	GLY42, **ILE43**,PHE47,GLU45,**HIS44**	IRIGENIN:H31 -:GLU45:O(2.082 A˚). GLU45:H - : IRIGENIN:O3(1.81 A˚). GLU45:H - : IRIGENIN:O4(2.29 A˚). **HIS44:HD1 - : IRIGENIN:O3(2.19 A˚). HIS44:HD1 - IRIGENIN:O4(1.89 A˚). IRIGENIN:H33 - ILE43:O (2.19 A˚). ILE43:H - : IRIGENIN:O6(2.43 A˚).**
Safranal	61041	-9.29	**HIS44**, TYR68, LEU46, GLY61, TYR36, LEU62, LEU59	TYR36:HH - :SAFRANAL:O1(1.99 A˚).
Emodin	3220	-8.43	ASP41, GLY42, **ILE43**, GLU45, **HIS44**	EMODIN:H30 - GLU45:OE1 (2.03 A˚). **ILE43**:H - : EMODIN:O2. (2.37 A˚). EMODIN:H28 - A:ASP41:O(1.84 A˚).
Tectorigenin	5281811	-8.23	GLU45,**ILE43**,GLY42,ASP41,**HIS44**	Tect:H34 - GLU45:OE1(1.91 A˚). **Tect:H29 -:ILE43:O(2.12 A˚). ILE43:H - :TECT:O3(2.05 A˚).**

Table S4: MM-PBSA calculations

**Summary**	**Values**
Van der Waal energy	-160.104 +/- 23.737 kJ/mol
Electrostatic energy	-8.257 +/- 8.986 kJ/mol
Polar solvation energy	39.374 +/- 14.616 kJ/mol
SAV energy	-92.616 +/- 17.234 kJ/mol
Binding energy	-221.602 +/- 35.657 kJ/mol

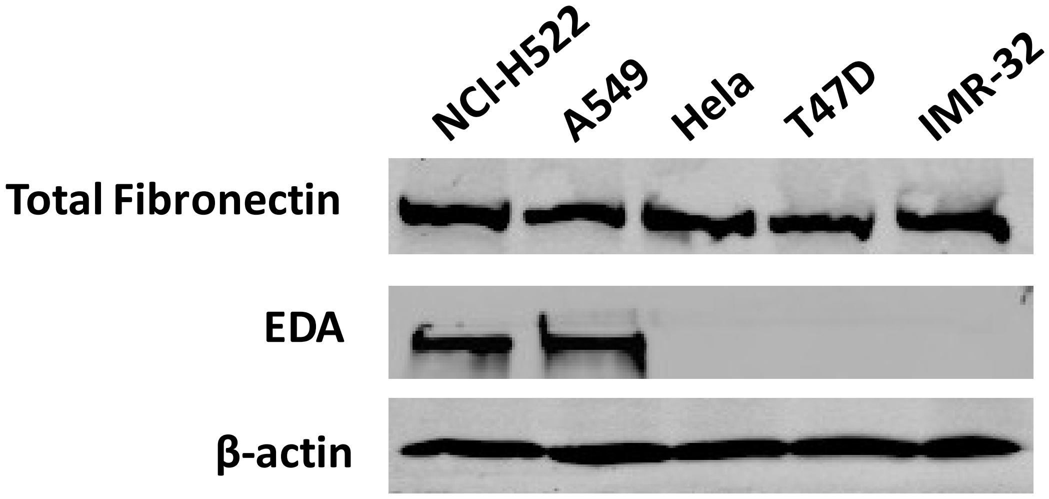

Figure S1: Western blotting analysis showing the expression of EDA in various cell lines.

a)

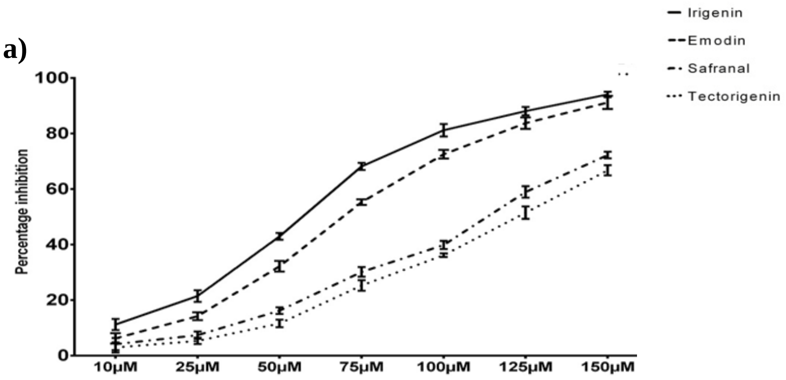

Compound	IC_{50} (µM) value A549
Irigenin	58.16±0.168
Emodin	68.76±0.235
Safranal	107.96±0.132
Tectorigenin	122.86±0.263

b)

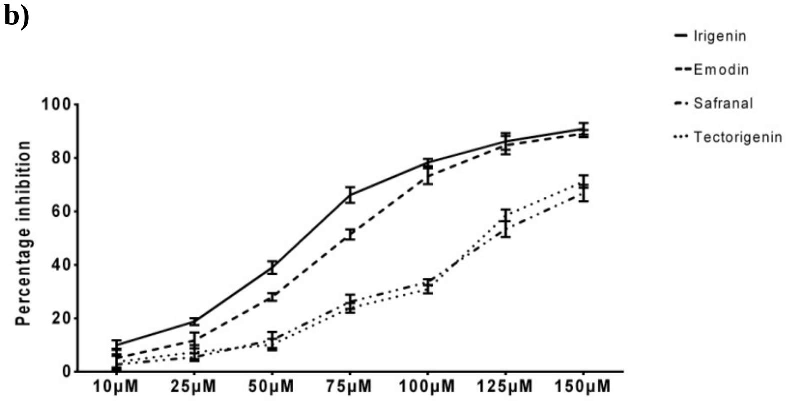

Compound	IC_{50} (µM) value NCI-H522
Irigenin	61 ± 0.133
Emodin	73.96 ± 0.268
Safranal	115.68 ± 0.163
Tectorigenin	118.52 ± 0.112

Figure S2: Anti-proliferative effect of shortlisted compounds on lung cancer cells (a) A549 and (b) NCI-H522. Cells were treated with the indicated concentrations (10-150 µM) of *Irigenin*, *Emodin*, *Safranal* and *Tectorigenin* respectively. After 24 h, the inhibition of cell proliferation was determined by MTT assay. Data represented as mean ±SD of results from three independent experiments. IC_{50} values of the selected compounds were evaluated by MTT assay.

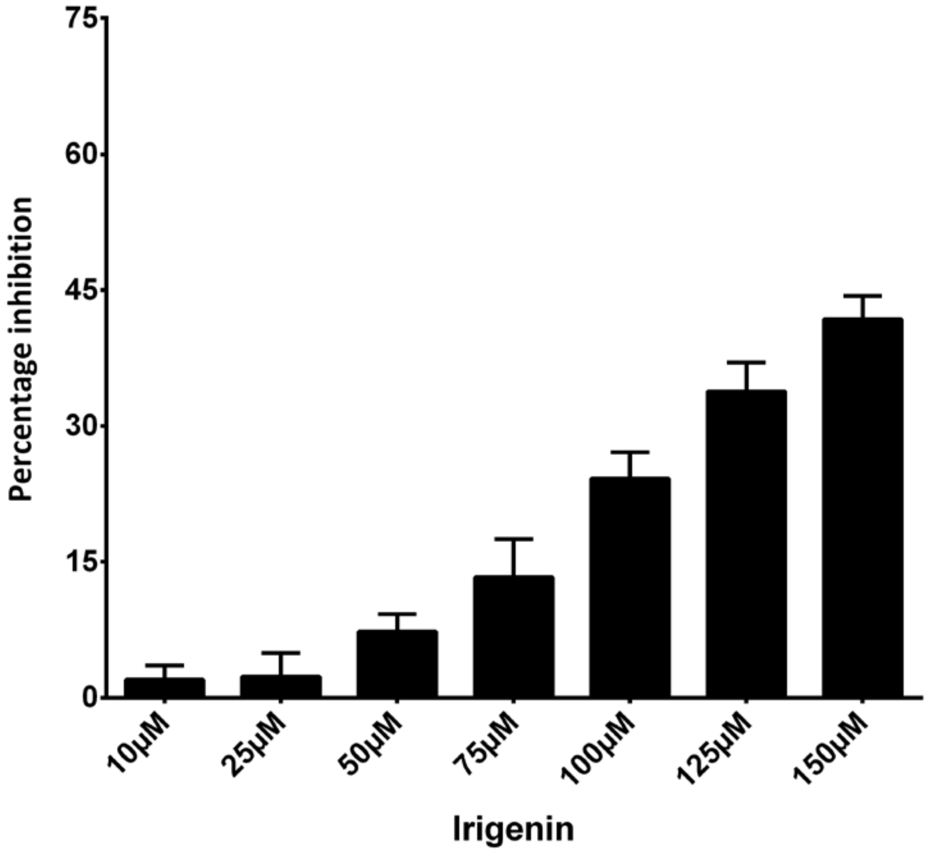

Figure S3: Anti-proliferative effect of *irigenin* on EDA negative, T47D cells. T47D cells were treated with the indicated concentrations (10-200 µM) of *irigenin.* After 24 h, the inhibition of cell proliferation was determined by MTT assay. Data represented as mean ±SD of results from three independent experiments.

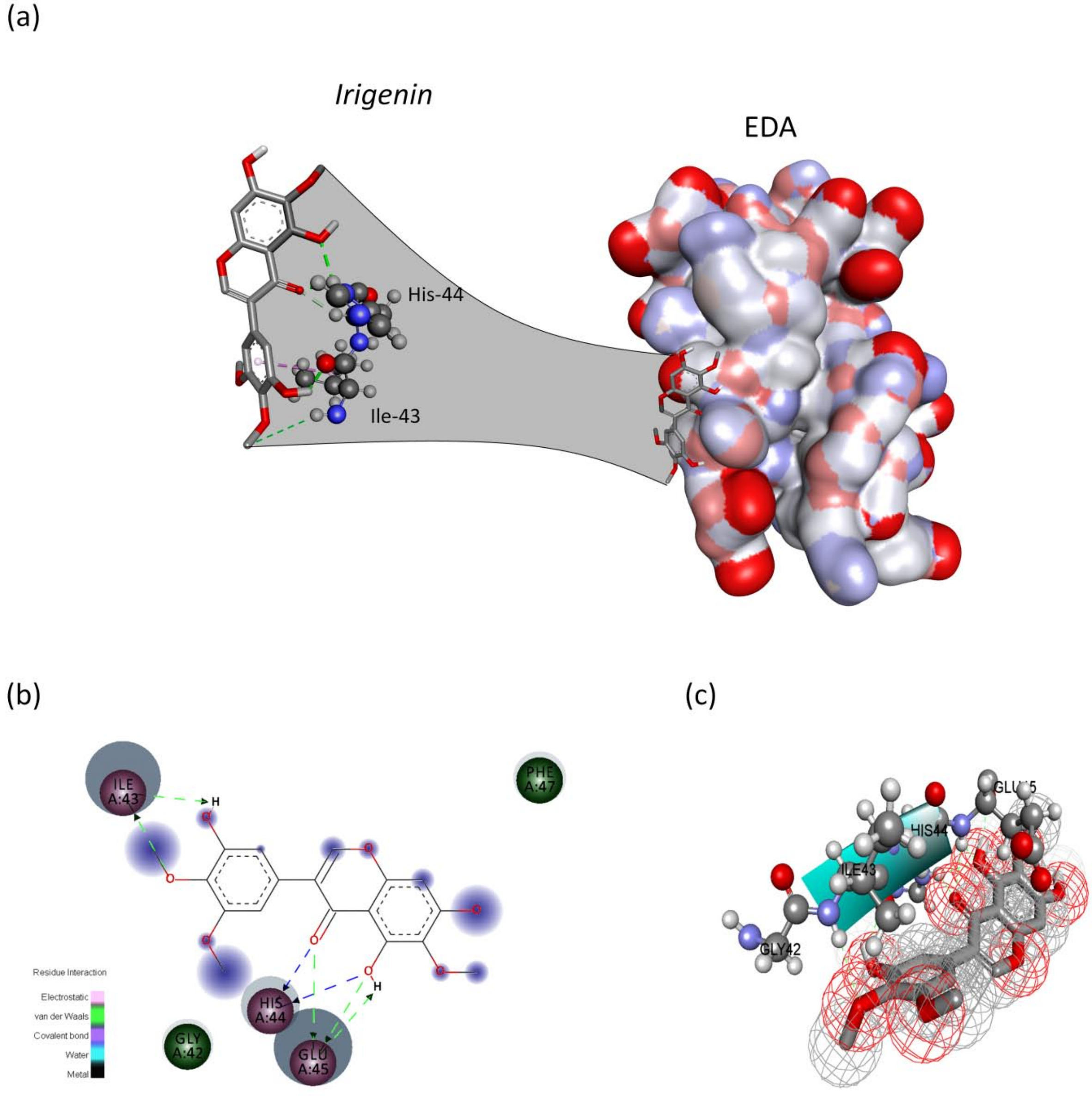

Figure S4: (a) The top binding pose obtained by molecular docking simulations. (b) The two dimensional representation of the top binding pose obtained using Discovery Studio 3.5, showing seven possible hydrogen bonds with ILE 43, HIS44 and GLU45. (c) Mapping of the atoms of *Irigenin* interacting with GLY42, ILE 43, HIS44 and GLU45 of C-C′ loop of EDA.

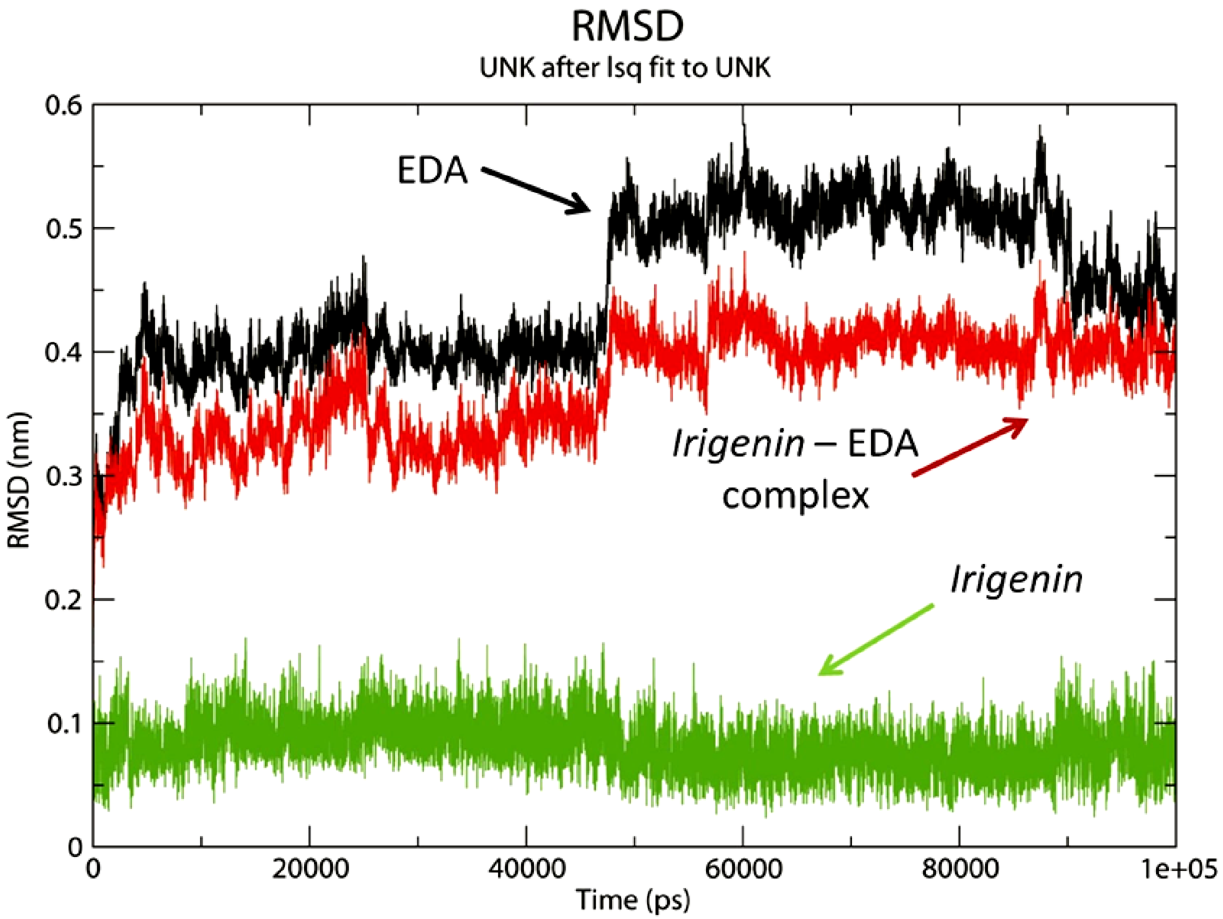

Figure S5: RMSD for complex of EDA-*irigenin*, EDA alone and *irigenin* alone calculated over all atoms as a function of time with respect to the starting conformation.

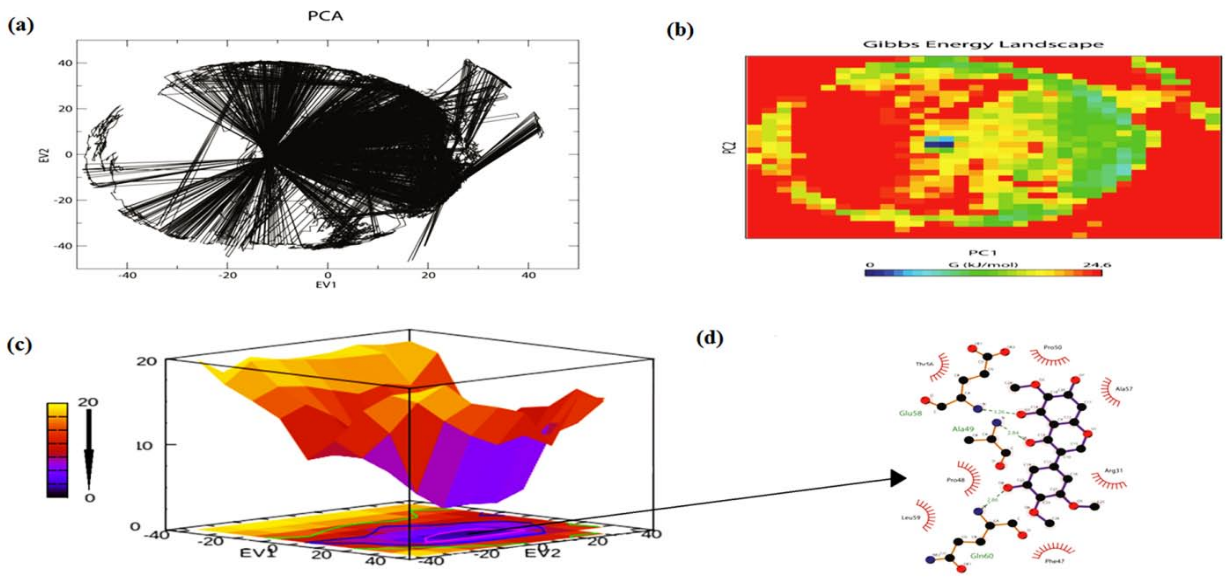

Figure S6: (a) Projection of the motion of the complex of the backbone of EDA and *Irigenin* along the first two principal eigenvectors. (b) Gibbs energy Landscape of first two principal eigenvectors using g_sham program. (c) Inverted Free energy landscape of projection of the motion along the first and second principal eigenvectors in phase space plotted for the backbone of EDA and *Irigenin*. (d) Representative hydrogen bond pattern of lowest energy conformation of simulated complex.

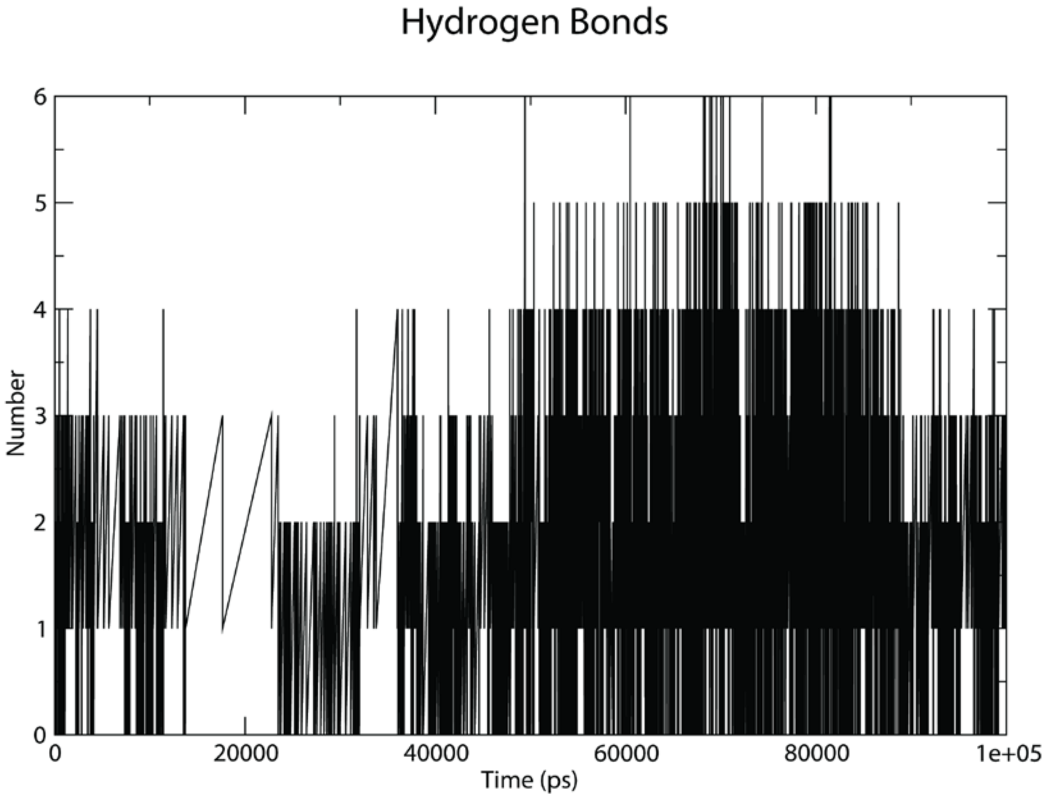

Figure S7: Hydrogen Bond pattern for EDA-*irigenin* complex over time.

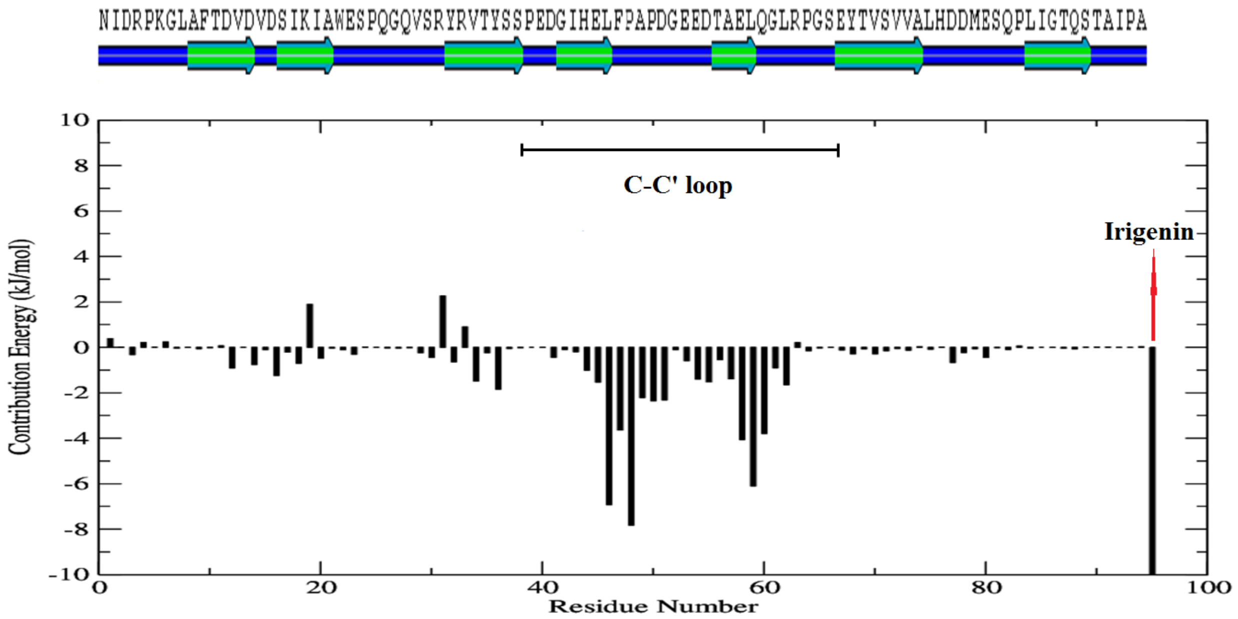

Figure S8: Energy per-residue decomposition of the *Irigenin*-EDA complex, showing the 40-60 amino acid region of the C-C′ loop to be most active.

Supplementary Movie: Molecular dynamics simulation of energy minimized *Irigenin*-EDA complex for 100 ns under GROMOS 43a1 force field. Each frame is retrieved at 100 ps interval.
